# Virtual Reality Enhances Gait in Cerebral Palsy: A Training Dose-Response Meta-Analysis

**DOI:** 10.3389/fneur.2019.00236

**Published:** 2019-03-26

**Authors:** Shashank Ghai, Ishan Ghai

**Affiliations:** ^1^Institute for Sports Science, Leibniz University Hannover, Hannover, Germany; ^2^Rsgbiogen, New Delhi, India

**Keywords:** cerebral palsy, gait, virtual reality, brain injury, rehabilitation

## Abstract

Virtual-reality-based training can influence gait recovery in children with cerebral palsy. A consensus concerning its influence on spatiotemporal gait parameters and effective training dosage is still warranted. This study analyzes the influence of virtual-reality training (relevant training dosage) on gait recovery in children with cerebral palsy. A search was performed by two reviewers according to Preferred Reporting Items for Systematic Reviews and Meta-Analyses (PRISMA) guidelines on nine databases: PEDro, EBSCO, PubMed, Cochrane, Web of Science, EMBASE, ICI, Scopus, and PROQUEST. Of 989 records, 16 studies involving a total of 274 children with cerebral palsy met our inclusion criteria. Eighty-eight percent of the studies reported significant enhancements in gait performance after training with virtual reality. Meta-analyses revealed positive effects of virtual-reality training on gait velocity (Hedge's *g* = 0.68), stride length (0.30), cadence (0.66), and gross motor function measure (0.44). Subgroup analysis reported a training duration of 20–30 min per session, ≤4 times per week across ≥8 weeks to allow maximum enhancements in gait velocity. This study provides preliminary evidence for the beneficial influence of virtual-reality training in gait rehabilitation for children with cerebral palsy.

## Introduction

Gait dysfunctions are prominent in children with cerebral palsy (1, 2). Reduction in gait velocity, cadence and stride length are common spatiotemporal gait characteristics exhibited by children with cerebral palsy (2). Recent experimental and review studies have reported the beneficial influence of virtual-reality training strategies to considerably influence gait performance in children with cerebral palsy (3, 4). According to Aminov et al. (5), virtual reality is a superior rehabilitative approach when compared with conventional therapeutic approaches. The authors suggest that this strategy can allow a patient to (re)learn motor skills while interacting with real-life scenarios in an ecological yet patient-centric manner (6).

The application of this intervention is dynamic as it allows real-time “multisensory” feedback of executed movement to both the performer and the medical practitioner. This further can simultaneously facilitate the motor planning and perception of the performer and allow the medical practitioner to monitor and control the complexity of the virtual-reality task/environment according to each performer's capability (7). Several underlying mechanisms through which virtual-reality training can facilitate motor rehabilitation have been reported. For instance, amplification of sensorimotor representation by augmented sensory feedback (8–12), enhancement of error feedback (13), reduction of cognitive load (14–17), reduction of musculoskeletal coactivation (18), increased arousal (19), and motivation (20) are few of the reasons by which virtual-reality training might enhance gait recovery (3, 4, 21). Moreover, neuroimaging studies have reported that training with virtual reality can facilitate recovery by instigating cortical reorganization (22) and neural plasticity (23, 24), thus suggesting a strong potential for virtual-reality-based training for recovering gait in children with cerebral palsy.

Recent systematic reviews have reported the beneficial effects of virtual-reality-based training on gait performance in children with cerebral palsy (3, 4). However, to the best of our knowledge, only one study has elucidated the influence of virtual-reality training on gait performance in children with cerebral palsy statistically, i.e., a meta-analysis (3). Chen et al. (3) performed a meta-analysis on eight studies and reported a positive effect size of 0.75 (0.34–1.16) on the ambulation function after training with virtual reality. Although the findings of this study are in line with previous reviews, there were certain limitations. Firstly, the authors did not explore the cause of heterogeneity observed in the analysis, i.e., *I*^2^ = 59%. Secondly, the authors did not describe the specific variables evaluated in the ambulation function, i.e., no information was provided as to what these enhancements were applicable on, for instance, gait velocity, stride length, etc. Thirdly, the authors included some studies in the analysis that, on re-evaluation, were found to not have evaluated any gait parameter at all.

In the present systematic review and meta-analysis, our aim is to develop a state of evidence defining the influence of virtual-reality training on spatiotemporal gait parameters in children with cerebral palsy. Moreover, the importance of determining training dosages in neurological rehabilitation has been emphasized in several studies (25–31). Therefore, as a secondary objective, this present review also aims to elucidate effective training dosages for virtual-reality-based gait training that could be incorporated by medical practitioners during gait rehabilitation for children with cerebral palsy.

## Methods Summary

This review and meta-analysis was performed according to PRISMA guidelines (32). A systematic search of literature was performed across nine academic databases. The inclusion criteria were as follows: (i) Randomized controlled trials (RCTs) and Controlled clinical trials (CCTs), (ii) virtual-reality interventions (any training duration and setting), (iii) spatiotemporal gait parameters evaluated, (iv) gross motor function and/or performance measure evaluated, (v) ≥4 PEDro score, (vi) children with cerebral palsy (age range: 6–18 years), (vii) peer-reviewed publications, and (viii) in English, German, Hindi, Punjabi, and Sanskrit languages. For a detailed method section, see the Supplementary Material.

### Quality and Risk of Bias Assessment

The quality of the reviewed studies was assessed using the PEDro scale by both the reviewers (33).

### Level of Evidence Assessment

A level of evidence analysis, i.e., strength of recommendation, was assigned to each outcome measure, i.e., gait velocity, stride length, cadence, stride width, and gross motor function measure. This assessment was combinedly based on the methodological quality and design of the evaluated studies (34).

### Data Analysis

A within-group, i.e., pre–post meta-analysis, approach was performed to develop a better quantitative interpretation of the virtual-reality intervention (35). The meta-analyses were conducted using CMA (Comprehensive meta-analysis V 2.0, USA). The meta-analyses evaluated the influence of virtual-reality training on gait velocity, cadence, stride length, stride width, and gross motor function measure score. An analysis for publication bias was performed by Duval and Tweedie's trim and fill procedure (36). The alpha level was set at 5%.

### Characteristics of Included Studies

**Figure 1:**
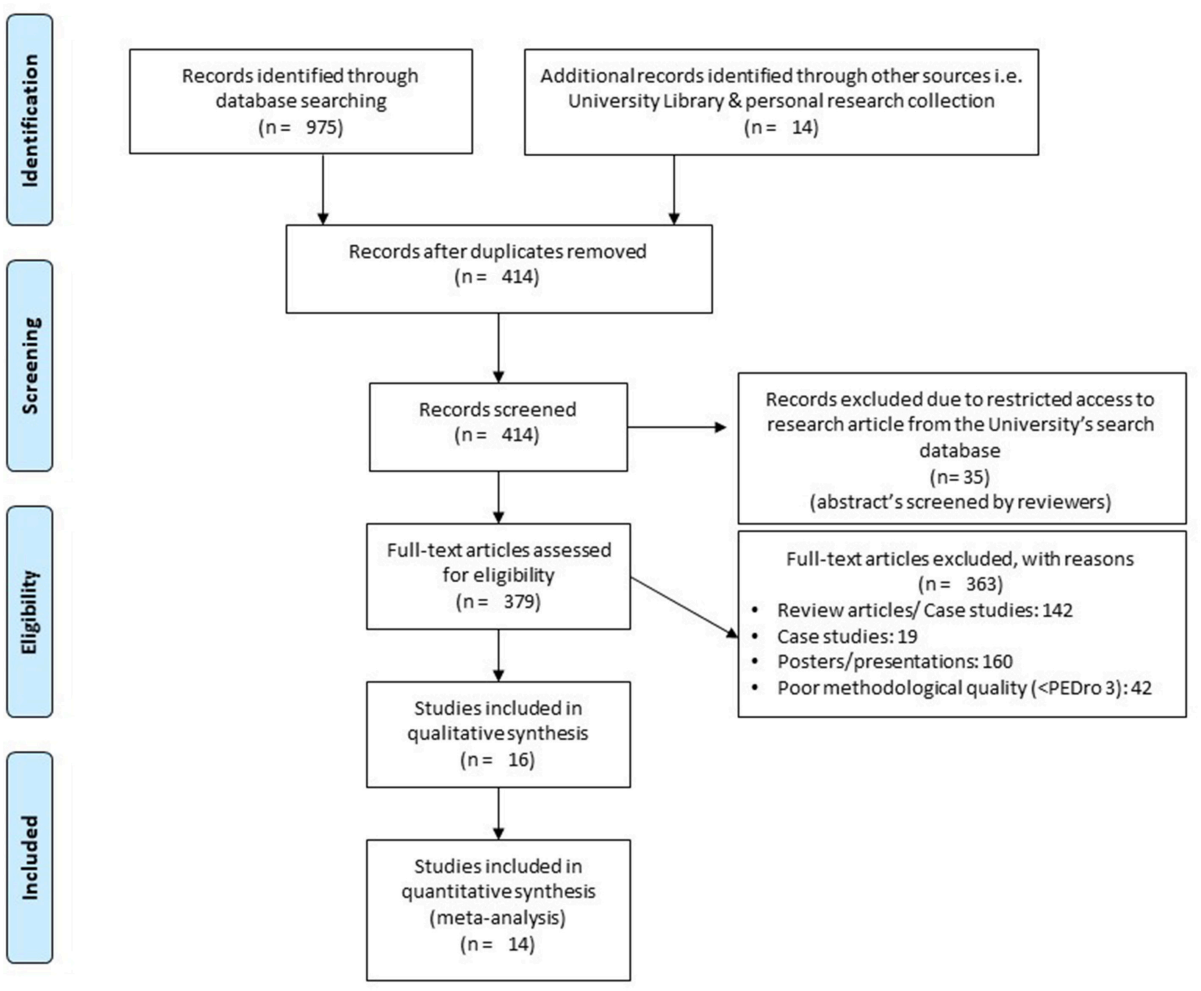
PRISMA flowchart for the inclusion of studies (32).

The initial search across the nine academic databases yielded a total of 989 studies, which, upon implementing the inclusion criteria, were reduced to 16 (Figure 1). Thereafter, both qualitative and quantitative data were extracted from all the studies (Supplementary Table 2). Of the 16 included studies, 5 were randomized controlled trials and 11 were controlled clinical trials.

#### Participants

A total of 274 participants were analyzed in the 16 incorporated studies. The included studies provided data on 120 females and 154 males. Descriptive statistics relating to the age (mean ± standard deviation, range) have been mentioned in Supplementary Table 3. In the included studies, three studies did not define the gender distribution (37–39).

#### Risk of Bias

**Figure 2:**
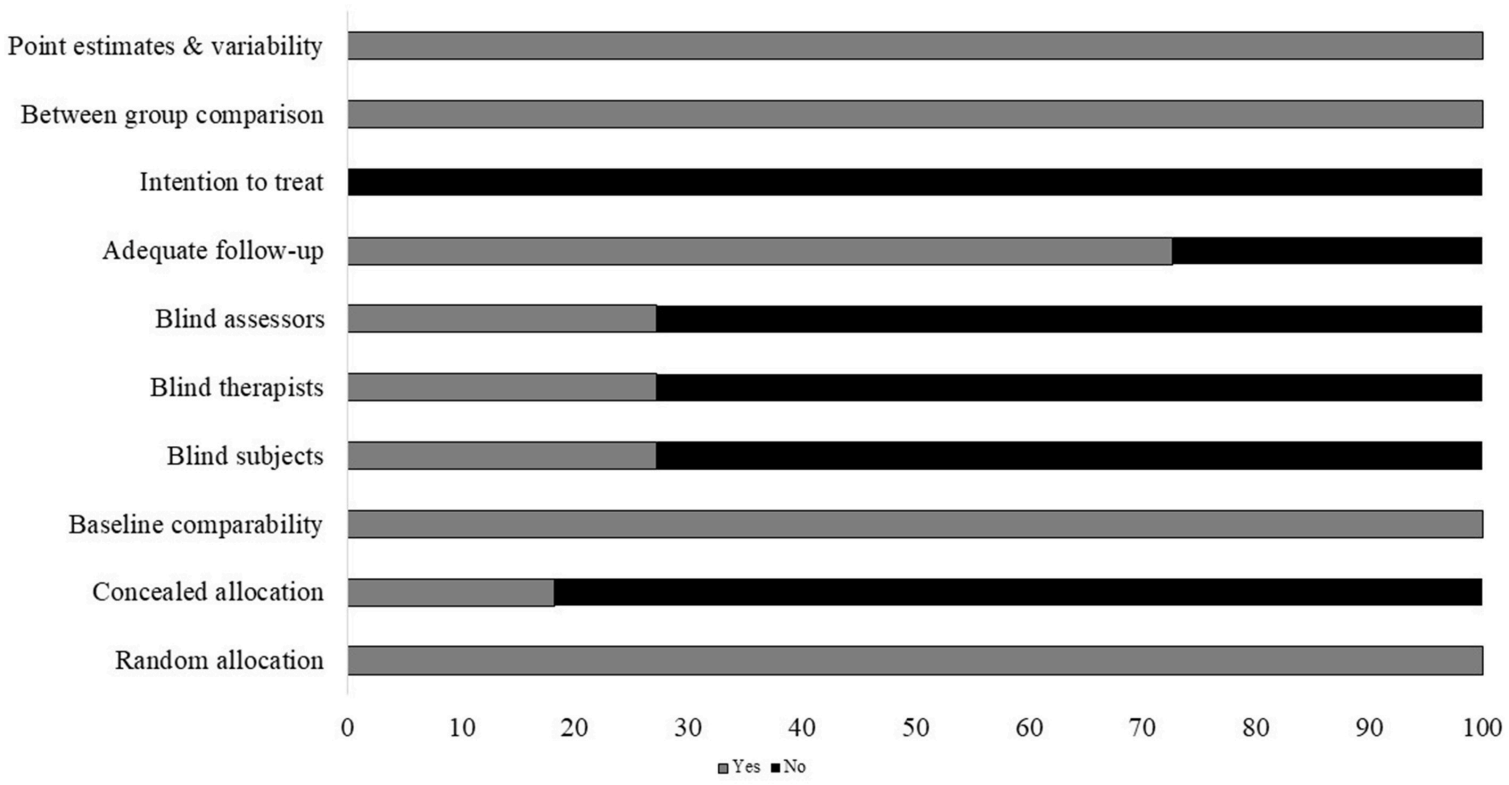
Risk of bias across studies (x axis: %).

Individual scores attained by the studies using the PEDro scale for each factor have been mentioned (Supplementary Table 4). The average PEDro score of the 18 included studies was computed to be (M ± S.D). 5.7 ± 1.4 out of 10, indicating, on average, a “good” quality of the studies. Here, one study scored 9 (40), one study scored 8 (41), three scored 7 (39, 42, 43), two scored 6 (44, 45), six scored 5 (37, 46–50), and three studies scored 4 (38, 51, 52). The risk of biasing across the studies has been illustrated in Figure 2. Individual scoring by the studies on each parameter has been mentioned in Supplementary Table 3.

**Figure 3:**
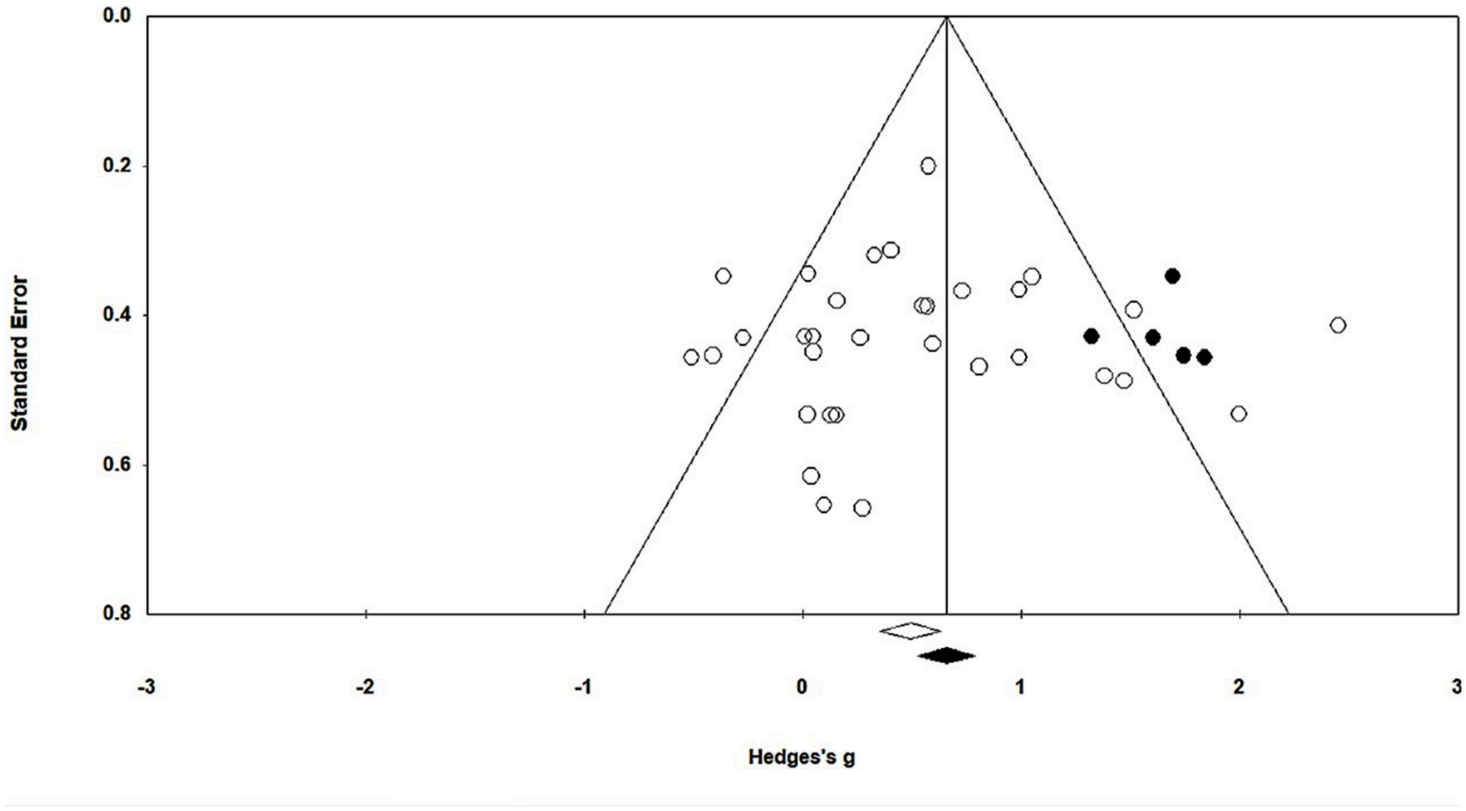
Trim and fill funnel plot for Hedge's g and standardized effect for each value in the meta-analysis. Each of the effect is represented in the plot as a circle. Funnel boundaries represent area where 95% of the effects are expected to lie if there were no publication biases. The vertical line represents the mean standardized effect of zero.

In this present study, publication bias was analyzed by Duval and Tweedie's trim and fill method. The graph plots the evaluated weighted effect size, i.e., Hedge's *g* values against standard error (Figure 3). Here, the absence of publication bias is determined by symmetrical distribution of the studies about the combined effect size. The trim and fill test elucidated any missing studies based on a fixed effect model in the present analysis. However, the method suggests that no studies are missing. Under the random effects model, the point estimate is 0.48 and the 95% confidence interval (CI) is 0.26–0.71 for the combined studies. Using trim and fill, the imputed point estimate is 0.66 and the 95% CI is 0.43–0.89.

### Level of Evidence

The analysis of level of evidence based on evidence-based nursing care guidelines revealed a “III Level of Evidence,” supporting the beneficial effects of virtual-reality training on gait and motor performance in children with cerebral palsy. This level of evidence was awarded to all the evaluated parameters and dose–response subgroup analyses. The appraisal of level of evidence score was based on the design and scoring of the included studies, i.e., controlled clinical trials.

## Outcomes

The current qualitative and quantitative evidence from the review suggests the beneficial effects of virtual-reality training on spatiotemporal gait parameters for children with cerebral palsy. Nine included studies reported significant enhancement in gait performance for children with cerebral palsy after virtual-reality training. Two studies reported no influence of virtual-reality training gait on spatiotemporal gait parameters (see Supplementary Table 3) (48, 50).

## Meta-Analysis Report

### Gait Velocity

**Figure 4:**
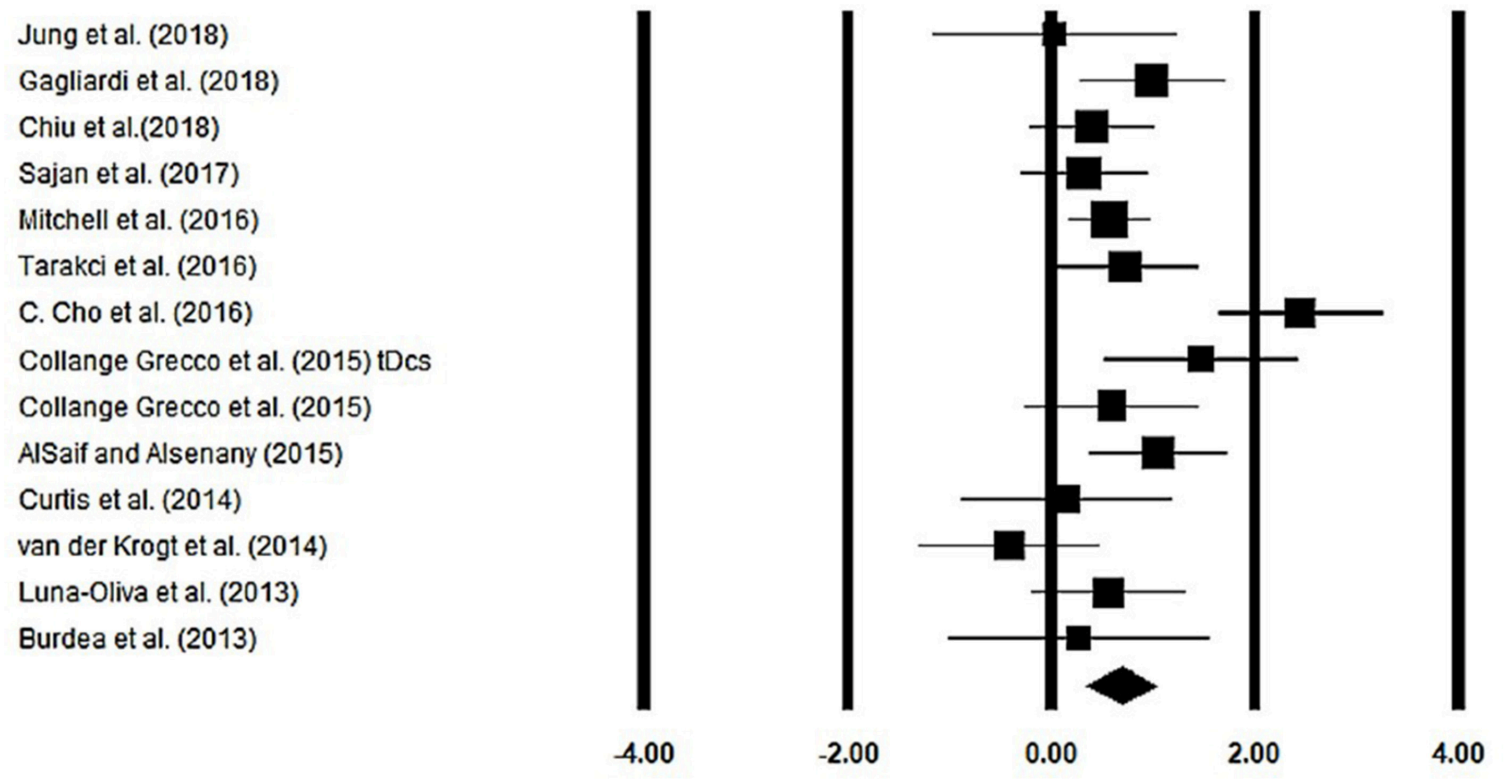
Forest plot illustrating individual studies evaluating the effects of virtual-reality training on gait velocity among children with cerebral palsy. Weighted effect sizes, Hedge's g (boxes), and 95% CI (whiskers) are presented, demonstrating repositioning errors for individual studies. The (Diamond) represents pooled effect sizes and 95% CI.

Gait velocity was assessed among 13 studies. Additional data were extracted from one study (40). The analysis of studies revealed (Figure 4) a *medium* effect size in the positive domain (*g* = 0.68, 95% CI: 0.35 to 1.01) with negligible heterogeneity (*I*^2^ = 13.1%, *p* > 0.05). In the included studies, only one study did not utilize a training intervention with virtual reality (50). Therefore, in a subsequent analysis, we removed this study and reperformed the analysis to elucidate the influence of virtual-reality training on gait velocity. The analysis of studies revealed (Supplementary Figure 1) a *large* effect size in the positive domain (*g* = 0.76, 95% CI: 0.44 to 1.07) with negligible heterogeneity (*I*^2^ = 10.7%, *p* > 0.05).

#### Session Length

20–30 min: A subgroup analysis was performed on nine studies. The analysis revealed a *large* effect size (Supplementary Figure 2) in the positive domain (*g* = 0.88, 95% CI: 0.51 to 1.24) with negligible heterogeneity (*I*^2^ = 12.1%, *p* > 0.05).
40–45 min: A subgroup analysis was performed on three studies. The analysis revealed a *medium* effect size (Supplementary Figure 3) in the positive domain (*g* = 0.26, 95% CI: −0.24 to 0.77) with no heterogeneity (*I*^2^ = 0%, *p* > 0.05).

#### Sessions per Week

≤4 sessions per week: A subgroup analysis was performed on six studies. The analysis revealed a *large* effect size (Supplementary Figure 4) in the positive domain (*g* = 0.78, 95% CI: 0.09 to 1.47) with negligible heterogeneity (*I*^2^ = 1.4%, *p* > 0.05).
≥5 sessions per week: A subgroup analysis was performed on six studies. The analysis revealed a *medium* effect size (Supplementary Figure 5) in the positive domain (*g* = 0.69, 95% CI: 0.42 to 0.97) with negligible heterogeneity (*I*^2^ = 2.4%, *p* > 0.05).

#### Number of Weeks of Training

≥8 weeks: A subgroup analysis was performed on four studies. The analysis revealed a *medium* effect size (Supplementary Figure 6) in the positive domain (*g* = 0.69, 95% CI: 0.25 to 1.13) with negligible heterogeneity (*I*^2^ = 2.0%, *p* > 0.05).
≤7 weeks: A subgroup analysis was performed on eight studies. The analysis revealed a *medium* effect size (Supplementary Figure 7) in the positive domain (*g* = 0.65, 95% CI: 0.35 to 0.94) with no heterogeneity (*I*^2^ = 0.69%, *p* > 0.05).

### Stride Length

**Figure 5:**
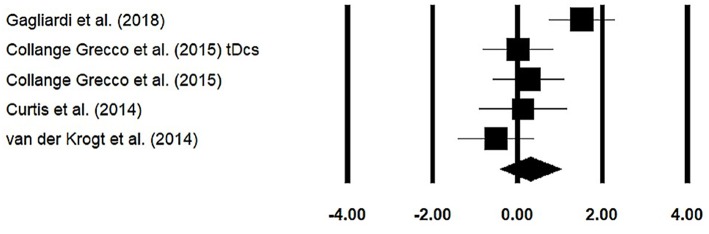
Forest plot illustrating individual studies evaluating the effects of virtual-reality training on stride length among children with cerebral palsy. Weighted effect sizes, Hedge's *g* (boxes), and 95% CI (whiskers) are presented, demonstrating repositioning errors for individual studies. The (diamond) represents pooled effect sizes and 95% CI.

Stride length was assessed among four studies. Additional data were extracted from one study (40). The analysis of the studies revealed (Figure 5) a *medium* effect size in the positive domain (*g* = 0.30, 95% CI: −0.40 to 1.01) with no heterogeneity (*I*^2^ = 0%, *p* > 0.05). In the included studies, only one study did not utilize a training intervention with virtual reality (50). Therefore, in a subsequent analysis, we removed this study and reperformed the analysis to elucidate the influence of virtual-reality training on stride length. The analysis of studies revealed (Supplementary Figure 8) a *medium* effect size in the positive domain (*g* = 0.50, 95% CI: −0.20 to 1.24) with no heterogeneity (*I*^2^ = 0%, *p* > 0.05).

### Cadence

Cadence was assessed among two studies. Additional data were extracted from one study (40). The analysis of studies revealed (Supplementary Figure 9) a *medium* effect size in the positive domain (*g* = 0.66, 95% CI: −0.52 to 1.84) with negligible heterogeneity (*I*^2^ = 10.8%, *p* > 0.05).

### Stride Width

**Figure 6:**
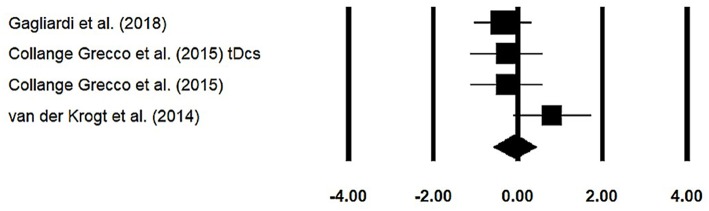
Forest plot illustrating individual studies evaluating the effects of virtual-reality training on stride width among children with cerebral palsy. Weighted effect sizes, Hedge's *g* (boxes), and 95% CI (whiskers) are presented, demonstrating repositioning errors for individual studies. The (diamond) represents pooled effect sizes and 95% CI.

Stride width was assessed among three studies. Additional data were extracted from one study (40). The analysis of studies revealed (Figure 6) a *small* effect size in the negative domain (*g* = −0.07, 95% CI: −0.57 to 0.43) with negligible heterogeneity (*I*^2^ = 4.5%, *p* > 0.05). In the included studies, only one study did not utilize a training intervention with virtual reality (50). Therefore, in a subsequent analysis, we removed this study and reperformed the analysis to elucidate the influence of virtual-reality training on stride width. The analysis of studies revealed (Supplementary Figure 10) a *small* effect size in the negative domain (*g* = −0.23, 95% CI: −0.53 to 0.06) with moderate heterogeneity (*I*^2^ = 0%, *p* > 0.05).

### Gross Motor Function Measure

**Figure 7:**
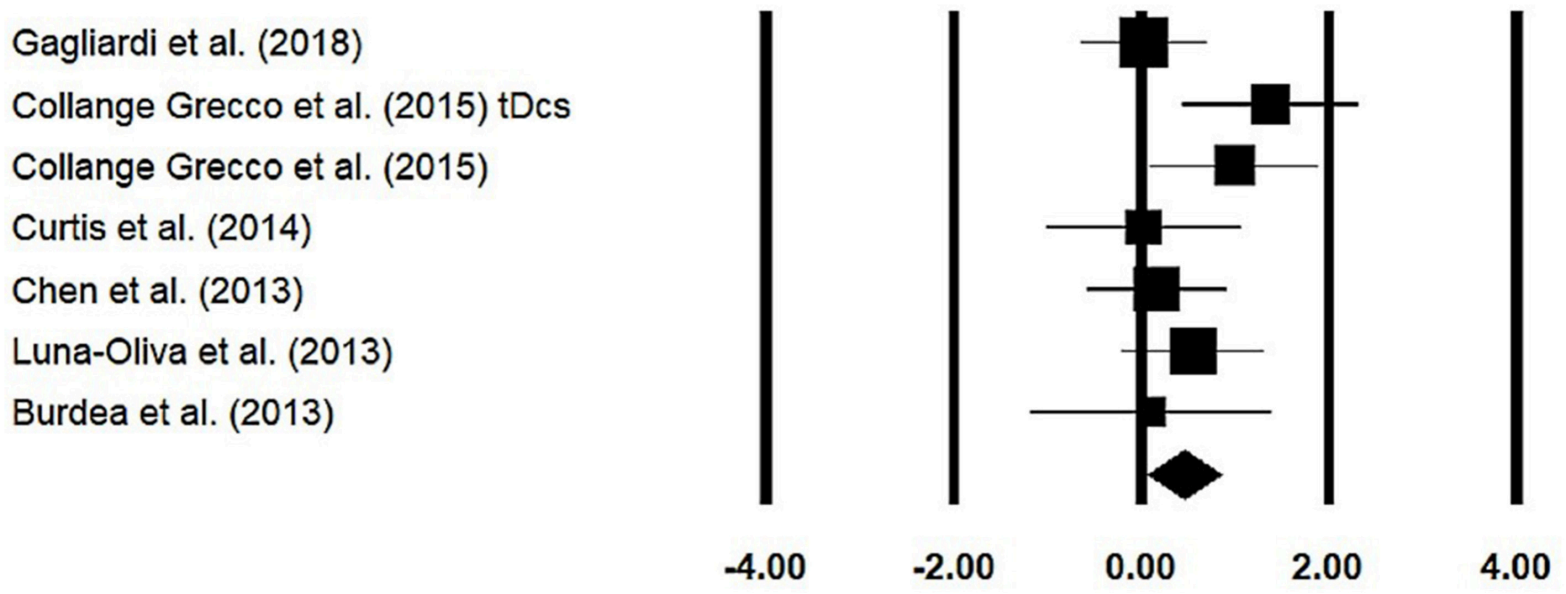
Forest plot illustrating individual studies evaluating the effects of virtual-reality training on gross motor function measure among children with cerebral palsy. Weighted effect sizes, Hedge's *g* (boxes), and 95% CI (whiskers) are presented, demonstrating repositioning errors for individual studies. The (diamond) represents pooled effect sizes and 95% CI.

Gross motor function measure was assessed among six studies. The analysis of studies revealed (Figure 7) a *medium* effect size in the positive domain (*g* = 0.44, 95% CI: 0.06 to 0.83) with negligible heterogeneity (*I*^2^ = 0.54%, *p* > 0.05).

#### Session Length

20–30 min: A subgroup analysis was performed on four studies. The analysis revealed a *medium* effect size (Supplementary Figure 11) in the positive domain (*g* = 0.56, 95% CI: 0.05 to 1.07) with negligible heterogeneity (*I*^2^ = 0.08%, *p* > 0.05).
40–45 min: A subgroup analysis was performed on two studies. The analysis revealed a *small* effect size (Supplementary Figure 12) in the positive domain (*g* = 0.14, 95% CI: −0.50 to 0.78) with no heterogeneity (*I*^2^ = 0%, *p* > 0.05).

## Discussion

The primary objective of this present systematic review and meta-analysis was to synthesize the current state of knowledge to determine the effects of virtual-reality training on spatiotemporal gait parameters in children with cerebral palsy. The findings from the current meta-analyses suggest a positive influence of virtual-reality training to enhance gait performance. Spatiotemporal parameters, i.e., gait velocity, cadence, and stride length, which are usually adversely affected in cerebral palsy (53), were enhanced after training with virtual reality, i.e., gait velocity (*g* = 0.76), stride length (*g* = 0.76), and cadence (*g* = 0.80).

Studies have suggested that virtual-reality training can facilitate motor performance by providing a performer with real-time “multisensory” feedback of the executed movement (19, 54, 55). Children with cerebral palsy have been reportedly associated with substantial deficits in sensory perception, which might affect their motor planning and performance (56). Here, the addition of different sensory modalities, for instance, auditory, visual, and proprioceptive feedback, could provide a “sensory deficit” patient with enriched knowledge of performance (movement amplitudes, relative limb position) and result (4, 19, 57–59).

Additionally, the enhancements in spatiotemporal gait parameters could be attributed to substantial changes in force, power, and kinematics at the ankle and knee joints (60). According to Chen et al. (46), virtual-reality-based training can substantially enhance isokinetic muscle strength and the amount of physical activity performed by children with cerebral palsy. This was also demonstrated in our analysis where gross motor function measure was enhanced (0.45) after training with virtual reality. In terms of movement kinematics, we presume that the explicit multisensory (i.e., visual–auditory–proprioceptive) feedback concerning the movement execution within the virtual environment could have allowed the patients to specifically time and control their movement patterns [see guidance hypothesis (61, 62)], thereby promoting a smooth movement pattern with reduced musculoskeletal co-contraction (18). In this review, several studies reported enhancement in gait kinematic scores (45, 50, 63, 64), which usually are adversely affected in children with cerebral palsy.

Furthermore, the patient-centered, closed-loop [tailored difficulty progression (65)] approach of virtual-reality training could be an additional reason for the superior influence of this rehabilitation intervention as compared to conventional approaches like resistance training (66), rhythmic auditory cueing (67), and robot-assisted training (68). Here, linking the individual performance measures concerning the motor restrictions and cognitive performance with the adaptive modulation of the task to be trained can provide adaptive mechanics in the virtual environment that might facilitate neuroplasticity (69). For instance, Xiao et al. (22) in a neuroimaging study reported the beneficial influence of virtual-reality-based training on motor planning and execution centers. The authors reported enhanced activations in primary, secondary motor, sensorimotor, and premotor cortices in stroke patients after virtual-reality training [also see (19, 70)]. Interestingly, the authors also reported hyperactivity in the ipsilesional somatosensory cortex with virtual-reality training (22). This enhanced activation in the somatosensory cortex of the affected hemisphere could be interpreted as unmasking of (pre)existent movement patterns (functional recovery *via* motor relearning) (70, 71).

Finally, we also elucidated specific virtual-reality training dosages that could be beneficially incorporated to attain maximum benefits in gait recovery in children with cerebral palsy. Fluet and Deutsch (72) had previously emphasized future studies for deducing training dosages in neurorehabilitation. The authors hypothesized that larger training dosages might account for more enhancements in motor recovery. However, as per the current findings of this meta-analysis, this was not the case. In terms of the length of training sessions, higher increments in spatiotemporal gait parameters were noted during training interventions lasting for 20–30 min as compared to 40–45 min of training (gait velocity: 0.88 vs. 0.26, gross motor function test: 0.56 vs. 0.14). Likewise, similar increments were noted for the number of sessions per week, i.e., ≤4 sessions allowed higher increments in gait velocity as compared to ≥5 weeks (0.84 vs. 0.65). In terms of the number of weeks of training, more number of weeks was observed to allow a greater influence on training, i.e., ≥8 weeks allowed better performance as compared to ≤7 weeks of training (0.83 vs. 0.65).

Two major limitations persisted in the present review. First, this study was not pre-registered in a prospective register for systematic reviews, such as PROSPERO. Second, a dose–response meta-analysis was performed for some variables with very few number of studies. This could raise concerns regarding the reliability of some outcome measures, i.e., incurring a type II error. We strongly recommend the reader to interpret the results with caution. Nevertheless, our findings are in line with previously published “high-quality” systematic reviews and meta-analyses that report positive or negligible effects of virtual-reality training on gait performance in children with cerebral palsy (3, 4). However, this present review extends the findings of these studies due to several reasons. Firstly, the present review incorporates a higher number of experimental studies that support our conclusion, i.e., 16 studies (274 participants) as compared to previously published review studies (3, 4). This large difference in the number of included studies could be attributed to the higher number of relevant academic databases searched (with multiple languages), i.e., nine, and the inclusion of controlled clinical trials. Secondly, this current review presents robust evidence to suggest training dosage with virtual reality for allowing enhancements in gait velocity and gross motor function test. Thirdly, this study provides evidence for the beneficial effects of virtual-reality training on generalized physical activity and motor output. It is important for the reader to consider that it is not our intention to disregard previously published reviews and meta-analyses. These reviews have addressed different factors for individuals with cerebral palsy (i.e., quality of life, arm recovery, and postural control), which was not the objective of the present review. Therefore, in our opinion, interpretations should be drawn simultaneously from all the reviews to develop a better understanding of the influence of virtual-reality-based training strategies for gait recovery after cerebral palsy. A III Level of Evidence supported the beneficial effects of virtual-reality-based training on gait performance. This weak level of evidence strongly warrants the need for multiple, high-quality, multicentered, randomized controlled trials to support the application of virtual-reality training on gait performance in children with cerebral palsy.

In conclusion, virtual-reality training facilitates gait performance in children with cerebral palsy. The present study reports the influence of virtual-reality-based training on the most commonly evaluated spatiotemporal gait outcomes; this shall allow the clinicians to better interpret the results with previous studies and other interventions and deduce practical implications for the benefit of children with cerebral palsy. The review, based on limited state of evidence, i.e., III Level of Evidence, suggests a training duration of at least 20–30 min, ≤4 times per week across ≥8 weeks.

## Author Contributions

SG conceptualized the study, carried out the systematic-review, statistical analysis, and wrote the paper. IG assisted in the systematic-review process and reviewed the manuscript.

### Conflict of Interest Statement

IG was employed by Rsgbiogen. The remaining author declares that the research was conducted in the absence of any commercial or financial relationships that could be construed as a potential conflict of interest.
