# Supplementary material for: Virtual Reality Enhances Gait in Cerebral Palsy: A Training Dose-Response Meta-Analysis
Source: Front Neurol. 2019 Mar 26;10:236. doi: 10.3389/fneur.2019.00236 (PMC6448032; doi:10.3389/fneur.2019.00236)
Supplement: Supplementary file 1 [file Table_1.DOCX]

**Virtual reality training enhances gait in cerebral palsy: A training dose-response meta-analysis**

Shashank Ghai^a^*, Ishan Ghai^b^

^a^Institute for Sports Science, Leibniz University Hannover, Germany

^b^Rsgbiogen, New Delhi, India

**Methods**

This review and meta-analysis was performed according PRISMA guidelines (Moher, Liberati, Tetzlaff, Altman, & Group, 2009). A checklist has been provided as Supplementary Table 1.

### **Data sources and search strategy**

Nine academic databases (Web of science, PEDro, EBSCO, Pubmed, Indian citation index, Scopus, Cochrane central register of controlled trials, EMBASE and PROQUEST) were searched from inception until July 2018. A sample PICOS search strategy for academic database EMBASE has been demonstrated in (Supplementary Table 2).

An inclusion criterion was established by two reviewers (S.G, I.G). The criteria for the studies were (i) Studies were either randomized controlled trials, cluster randomized controlled trials or controlled clinical trials (ii) Evaluated virtual-reality training interventions (any training duration, treatment setting) (iii) Evaluated spatiotemporal gait parameters (descriptive data for gait velocity and/or cadence and/or stride length and/or stride time and/or single/double-limb support duration)(Gasq et al., 2017) (v) Evaluated subjective Gross motor function and/or performance measure (with descriptive data)(Hanna, Bartlett, Rivard, & Russell, 2008) (vi) Methodological scoring ≥4 points on PEDro quality scale (studies scoring <3 considered of “poor” quality with high risk of biasing excluded) (Elkins, Herbert, Moseley, Sherrington, & Maher, 2010) (vii) Studies performed on children with cerebral palsy (age: 6-18 years, any type, any comorbidity) (viii) Published in peer-reviewed academic journals or conference proceedings (un-published “grey” literature not included) (ix) Studies published in English, German, Hindi, Punjabi and Sanskrit languages.

The two reviewers (S.G, I.G) duplicated both the study selection, and the data extraction procedure. After selection of articles, following data were extracted from each study i.e. author, journal name, publication year, selection criteria for participants, total sample size, description of the participants (gender, age, health status, nature of morbidity, comorbidities), applied treatment intervention, details of virtual reality interventions, treatment interventions for the control group, dual-task application (if any), outcome measures, results, conclusions and special notes by authors. The data were then summarized and tabulated (Supplementary Table 2). In cases where quantitative data was not presented in the manuscript. The reviewers (S.G, I.G) made attempts to contact respective corresponding authors for data.

### **Quality and risk of bias assessment**

The quality of the reviewed studies was assessed using the PEDro scale by both the reviewers.(de Morton, 2009) The interpretation of methodological score out of 10 was that studies which scored between 9-10 were considered of “excellent”, 6-8 of “good”, 4-5 of “fair”, and <3 of “poor” quality.(Teasell, 2008)

### **Data Analysis**

A within-group i.e. pre-post meta-analysis approach was performed to develop a better quantitative interpretation of the virtual-reality intervention.(Borenstein, Hedges, Higgins, & Rothstein, 2010) The meta-analyses were conducted using CMA (Comprehensive meta-analysis V 2.0, USA). The data in this analysis was distributed and separately analyzed for each variable, such as gait velocity, step width, stride length, cadence and Gross motor function measure. Here, the use of either random or fixed effect meta-analysis was dependent upon the presence/absence of heterogeneity during the group analysis.(J. P. Higgins & Green, 2011) Moreover, forest plots with 95% confidence intervals were plotted. The effect sizes were adjusted and reported as weighted Hedge’s g.(Cumming, 2013) The thresholds for the interpretation of weighted effect sizes are as follows: an effect size of 0.2 is considered as a small effect, 0.5 as a medium effect and 0.8 as a large effect.(Cohen, 1988) Further, heterogeneity between the studies was computed using I^2^ statistics.(Cohen, 1988; J. P. T. Higgins, Thompson, Deeks, & Altman, 2003) The interpretation of heterogeneity via I^2^ statistics is as follows: 0-25% is considered as negligible heterogeneity, 25%-75% as moderate heterogeneity and ≥75% as substantial heterogeneity, respectively. In cases where substantial heterogeneity was observed sensitivity analysis were performed to elucidate the cause of heterogeneity.(Cooper, Hedges, & Valentine, 2009) In this analysis, the results were compared by either including or excluding results from studies that used inadequate randomization methods.

Details of weighted effect size, 95% confidence intervals, significant and heterogeneity have been reported for each variable. Additionally, an analysis for publication bias was performed by Duval and Tweedie's trim and fill procedure.(Sue & Richard, 2000) This method involves imputation of the asymmetric studies from the left side to locate the unbiased effect and then re-fills the plot by reinserting the trimmed studies on the left and their imputed counterparts on the right to the mean effect.(Borenstein, 2005) The alpha level was set at 5%.

| **Section/topic** | **#** | **Checklist item** | **Reported on page #** |
| --- | --- | --- | --- |
| **TITLE** | | |  |
| Title | 1 | Identify the report as a systematic review, meta-analysis, or both. | 1 |
| **ABSTRACT** | | |  |
| Structured summary | 2 | Provide a structured summary including, as applicable: background; objectives; data sources; study eligibility criteria, participants, and interventions; study appraisal and synthesis methods; results; limitations; conclusions and implications of key findings; systematic review registration number. | 2 |
| **INTRODUCTION** | | |  |
| Rationale | 3 | Describe the rationale for the review in the context of what is already known. | 3-6 |
| Objectives | 4 | Provide an explicit statement of questions being addressed with reference to participants, interventions, comparisons, outcomes, and study design (PICOS). | 6 |
| **METHODS** | | |  |
| Protocol and registration | 5 | Indicate if a review protocol exists, if and where it can be accessed (e.g., Web address), and, if available, provide registration information including registration number. | - |
| Eligibility criteria | 6 | Specify study characteristics (e.g., PICOS, length of follow-up) and report characteristics (e.g., years considered, language, publication status) used as criteria for eligibility, giving rationale. | 4 |
| Information sources | 7 | Describe all information sources (e.g., databases with dates of coverage, contact with study authors to identify additional studies) in the search and date last searched. | 4 |
| Search | 8 | Present full electronic search strategy for at least one database, including any limits used, such that it could be repeated. | Table 1, 4 |
| Study selection | 9 | State the process for selecting studies (i.e., screening, eligibility, included in systematic review, and, if applicable, included in the meta-analysis). | 4-5 |
| Data collection process | 10 | Describe method of data extraction from reports (e.g., piloted forms, independently, in duplicate) and any processes for obtaining and confirming data from investigators. | 4-5 |
| Data items | 11 | List and define all variables for which data were sought (e.g., PICOS, funding sources) and any assumptions and simplifications made. | 4-5 |
| Risk of bias in individual studies | 12 | Describe methods used for assessing risk of bias of individual studies (including specification of whether this was done at the study or outcome level), and how this information is to be used in any data synthesis. | 6 |
| Summary measures | 13 | State the principal summary measures (e.g., risk ratio, difference in means). | 5 |
| Synthesis of results | 14 | Describe the methods of handling data and combining results of studies, if done, including measures of consistency (e.g., I^2^) for each meta-analysis. | 5 |

Page 1 of 2

| **Section/topic** | **#** | **Checklist item** | **Reported on page #** |
| --- | --- | --- | --- |
| Risk of bias across studies | 15 | Specify any assessment of risk of bias that may affect the cumulative evidence (e.g., publication bias, selective reporting within studies). | 6 |
| Additional analyses | 16 | Describe methods of additional analyses (e.g., sensitivity or subgroup analyses, meta-regression), if done, indicating which were pre-specified. | - |
| **RESULTS** | | |  |
| Study selection | 17 | Give numbers of studies screened, assessed for eligibility, and included in the review, with reasons for exclusions at each stage, ideally with a flow diagram. | 6 |
| Study characteristics | 18 | For each study, present characteristics for which data were extracted (e.g., study size, PICOS, follow-up period) and provide the citations. | 5-6 |
| Risk of bias within studies | 19 | Present data on risk of bias of each study and, if available, any outcome level assessment (see item 12). | 5-6 |
| Results of individual studies | 20 | For all outcomes considered (benefits or harms), present, for each study: (a) simple summary data for each intervention group (b) effect estimates and confidence intervals, ideally with a forest plot. | 6-8 |
| Synthesis of results | 21 | Present results of each meta-analysis done, including confidence intervals and measures of consistency. | 6-8 |
| Risk of bias across studies | 22 | Present results of any assessment of risk of bias across studies (see Item 15). | Table 3, 6 |
| Additional analysis | 23 | Give results of additional analyses, if done (e.g., sensitivity or subgroup analyses, meta-regression [see Item 16]). | 6-8 |
| **DISCUSSION** | | |  |
| Summary of evidence | 24 | Summarize the main findings including the strength of evidence for each main outcome; consider their relevance to key groups (e.g., healthcare providers, users, and policy makers). | 8-13 |
| Limitations | 25 | Discuss limitations at study and outcome level (e.g., risk of bias), and at review-level (e.g., incomplete retrieval of identified research, reporting bias). | 13 |
| Conclusions | 26 | Provide a general interpretation of the results in the context of other evidence, and implications for future research. | 13 |
| **FUNDING** | | |  |
| Funding | 27 | Describe sources of funding for the systematic review and other support (e.g., supply of data); role of funders for the systematic review. | - |

Table 2 Sample search strategy EMBASE according to PICOS model (P: Problem/Patient/Population, I: Intervention, C: Comparison, O: Outcome, S: Type of study)

| **PICOS** | **DATABSE** | **EMBASE** |
| --- | --- | --- |
|  | **DATE** | **10/07/2018** |
|  | **STRATEGY** | **#1 AND #2 AND #3 AND #4 AND #5 AND #6** |
| **P** | **#1** | ("CP" OR "Cerebral Palsy" OR "Cerebral Palsy athetoid" OR "Cerebral Palsy congenital" OR "Cerebral Palsy Diplegic infantile" OR "Cerebral Palsy dyskinetic" OR "Cerebral Palsy dystonic-rigid" OR "Cerebral Palsy hypotonic" OR "Cerebral Palsy mixed" OR "Cerebral Palsy monoplegic, infantile" OR "Cerebral Palsy quadriplegic infantile" OR "Cerebral Palsy Rolandic type" OR "Cerebral Palsy Spastic" OR "Congenital Cerebral Palsy" OR "diplegia-spastic" OR "Diplegic infantile cerebral palsy" OR "infantile cerebral palsy-diplegic" OR "infantile cerebral palsy-monoplegic" OR "infantile cerebral palsy-quadriplegic" OR "little disease" OR "little’s disease" OR "Monoplegic cerebral palsy" OR "Monoplegic infantile cerebral palsy" OR "Quadriplegic infantile cerebral palsy" OR "Spastic diplegia")/de OR (CP OR Cerebral Palsy OR Cerebral Palsy athetoid OR Cerebral Palsy congenital OR Cerebral Palsy Diplegic infantile OR Cerebral Palsy dyskinetic OR Cerebral Palsy dystonic-rigid OR Cerebral Palsy hypotonic OR Cerebral Palsy mixed OR Cerebral Palsy monoplegic, infantile OR Cerebral Palsy quadriplegic infantile OR Cerebral Palsy Rolandic type OR Cerebral Palsy Spastic OR Congenital Cerebral Palsy OR diplegia-spastic OR Diplegic infantile cerebral palsy OR infantile cerebral palsy-diplegic OR infantile cerebral palsy-monoplegic OR infantile cerebral palsy-quadriplegic OR little disease OR little’s disease OR Monoplegic cerebral palsy OR Monoplegic infantile cerebral palsy OR Quadriplegic infantile cerebral palsy OR Spastic diplegia):ti,ab |
| **I** | **#2** | (‘Virtual reality’ OR ‘Virtual reality exposure therapy’ OR ‘Virtual reality immersion therapy’ OR ‘Virtual reality therapy’ OR ‘VR’ OR ‘real-world video recordings’ OR ‘virtual reality training’ OR ‘virtual reality posture training’ OR ‘Virtual reality balance training’ OR ‘Virtual reality gait training’)/de OR (Virtual reality OR Virtual reality exposure therapy OR Virtual reality immersion therapy OR Virtual reality therapy OR VR OR real-world video recordings OR virtual reality training OR virtual reality posture training OR Virtual reality balance training OR Virtual reality gait training)ti,ab |
| **C** | **n/a** | n/a |
| **O** | **#3** | (‘walking’ OR ‘gait’ OR ‘locomotion’ OR ‘range of motion’ OR ‘ROM’ OR ‘ambulation’ OR ‘mobility’ OR ‘treadmill gait’ OR ‘balance’ OR ‘stability’ OR ‘stride’ OR ‘gait training’ OR ‘gait rehabilitation’)/de OR (walking OR gait OR locomotion OR range of motion OR ROM OR ambulation OR mobility OR treadmill gait OR balance OR stability OR stride OR gait training OR gait rehabilitation);ti,ab |
| **S** | **#4** | (‘intervention study’ OR ‘cohort analysis’ OR ‘longitudinal study’ OR ‘cluster analysis’ OR ‘crossover trial’ OR ‘cluster analysis’ OR ‘randomized trial’ OR ‘major clinical study’)/de OR (longitudinal OR cohort OR crossover trial OR cluster analysis OR randomized trial OR clinical trial OR controlled trial);ti,ab |
|  | **#5** | (‘rehabilitation’ OR ‘treatment’ OR ‘rehab’ OR ‘management’ OR ‘therapy’ OR ‘physiotherapy’ OR ‘physical therapy’ OR ‘prevention’ OR ‘risk prevention’)/de OR (rehabilitation OR treatment OR rehab OR management OR therapy OR physiotherapy OR physical therapy OR prevention OR risk prevention);ti,ab |
|  | **#6** | (‘age groups’ OR ‘children’ OR ‘adolescent’ OR ‘young’ OR ‘elderly’ OR ‘old’ AND (‘gender’ OR ‘male’ OR ‘female’)/de OR (age groups OR children OR adolescent OR young OR elderly OR old AND (gender OR male OR female));ti;ab |

Table 3 Effects of virtual reality training on spatiotemporal gait parameters in children with cerebral palsy

| **Author** | **Type of morbidity** | **Sample age: (M ± S.D)** | **Assessment** | **Training modalities** | **Training duration** | **Outcome** |
| --- | --- | --- | --- | --- | --- | --- |
| Levac et al. (2018) | Spastic and mixed | Exp: 2F, 3M (12±2.7)  Ct: 3F, 3M (13.3±3.0) | GMFT and 6-MWT | Exp: VR gait training with active video gaming with Xbox Kinect  Ct: Activity video gaming with Xbox Kinect | Session: 30 minutes, days: 5 | No effects on GMFT and 6-MWT in Exp. |
| Gagliardi et al. (2018) | Spastic and mixed | 6F, 10M (11.0±2.4) | Stance time, step width, stride length, stride time, GV, maximum ankle flexion-extension power, minimum ankle flexion-extension swing, ROM ankle flexion-extension stance/swing, knee flexion initial contact, minimum flexion-extension at knee, ROM for flexion-extension at knee, minimum flexion-extension hip, ROM flexion-extension at hip, ROM abduction-adduction at hip, ROM for pelvic tilt, ROM for pelvic obliquity, functional assessment questionnaire and GMFT | VR gait training in Gait Realtime Analysis Interactive Lab | Session: 30 minutes, days/week: 5, weeks: 4 | Significant enhancement in stride length, GV, GMFT, kinematics of hip, pelvis and ankle joint after training with VR. |
| Chiu, Ada, and Lee (2018) | Spastic and mixed | 9F, 11M (8.7±2.4) | Strength (ankle dorsiflexion, plantarflexion, knee extension), GV: 6-MWT, 10-MWT (preferred speed, fast), assistance to participation scale | VR gait training with Wii Fit U device | Session: 30 minutes, days/week: 3, weeks: 8 | Significant enhancement in Strength (ankle dorsiflexion, plantarflexion, knee extension), GV: 6-MWT, 10-MWT (preferred speed, fast) after training with VR.  Enhancement in assistance to participation scale. |
| Jung, Song, Kim, Lee, and Lee (2018) | Spastic and diplegia | 2F, 2M (9.5±1.0) | TUG, Paediatric balance scale, functional mobility scale, 6-MWT and Selective Motor Control scoring | VR training using Xbox Kinect | Session: 40 minutes, days/week: 3, weeks: 4 | Significant enhancement in TUG, Pediatric balance scale, functional mobility scale, 6-MWT and Selective Motor Control scoring after training with VR. |
| Sajan, John, Grace, Sabu, and Tharion (2017) | Spastic and mixed | Exp: 4F, 5M (10.6±3.7)  Ct: 5F, 5M (12.4±4.9) | Centre of pressure sway (eyes open/closed), GV, gait endurance, box & block test, paediatric berg balance scale, QUEST, TVPS score | Exp: VR gait training with Wii  Ct: Conventional physiotherapy | Session: 45 minutes, days/week: 6, weeks: 3 | Significant enhancement in GV, endurance, TVPS, QUEST score, paediatric berg balance score, and box and block test in Exp as compared to Ct.  Significant reduction in Centre of pressure sway (eyes open/closed) in Exp as compared to Ct. |
| Tarakci, Ersoz Huseyinsinoglu, Tarakci, and Razak Ozdincler (2016) | Spastic and mixed | Exp: 5F, 10M (10.4±2.6)  Ct: 6F, 9M (10.5±2.7) | FRT, sit to stand test, TUG, 10-MWT (sec), 10-stair climbing test, Wee-FIM, paediatric functional independence measure | Exp: VR gait training with Wii fit  Ct: Conventional balance training | Session: 20 minutes, days/week: 2, weeks: 12 | Significant enhancement in Wee-FIM, paediatric functional independence measure in Exp as compared to Ct.  Significant enhancement in FRT, sit to stand test, TUG, 10-MWT (sec), 10-stair climbing test performance in Exp after training with VR. |
| Mitchell, Ziviani, and Boyd (2016) | Spastic and hemiplegic | Exp: 25F, 26M (11.3±2.4)  Ct: 24F, 26M (11.4±2.6) | 6-MWT, step counts, functional strength, Mobility Questionnaire 28 item, assessment of Life Habits recreational domain and moderate to vigorous physical activity | Exp: VR training  Ct: Conventional physiotherapy | Session: 30 minutes, days/week: 6, weeks: 20 | Significant enhancement in functional strength, and 6-MWT after training with VR training. |
| Cho, Hwang, Hwang, and Chung (2016) | Spastic and diplegic | Exp: 20 participants (6-10)  Ct: 20 participants (6-10) | Movement Assessment Battery for Children-2, manual dexterity, balance score, aiming and catching score, Bruininks-Oseretsky test of motor proficiency subtest, and GV: 1-MWT | Exp: VR training with Wii  Ct: no training | Session: 30 minutes, days/week: 3, weeks: 8 | Significant enhancements in Movement Assessment Battery for Children-2, manual dexterity, balance score, aiming and catching score, Bruininks-Oseretsky Test of Motor Proficiency subtest, and GV in Exp as compared to Ct. |
| AlSaif and Alsenany (2015) | Spastic and mixed | Exp: 9 participants (10.2±3.4)  Ct: 9 participants (9.4±3.8) | Strength bilateral (knee extension, flexion), GMFT (standing, walking, running, jumping %), GV: 10-MWT, 2-MWT | Exp: VR gait training with treadmill  Ct: Treadmill training | Session: 20 minutes, days/week: 7, weeks: 12 | Significant enhancements in bilateral knee strength, 10-MWT, 2-MWT and GMFT (standing%) in Exp as compared to Ct. |
| Collange Grecco et al. (2015) | Diparesis | Exp I: 4F, 6M (8.2±1.6)  Exp II: 5F, 5M (8.8±1.1) | GV, cadence, stride length, step length, step width, stance phase (%), GPS, Gait variation score: pelvic (obliquity, rotation, tilt), hip (flexion-extension, abduction-adduction, rotation), knee (flexion-extension), ankle(dorsiflexion-plantarflexion), foot progression, motor evoked potential, social function and GMFT | Exp I: VR training with anodal transcranial direct current stimulation  Exp II: VR training with sham stimulation | Session: 20 minutes, days/week: 5, weeks: 2 | Exp I: Significant enhancement in GV, cadence, GMFT and motor evoked potential.  Exp II: Significant enhancement in GV and GFMT. |
| Curtis, Bencke, and Mygind (2014) | Spastic and mixed | 3F, 3M (9.3±2.3) | GV, step length, 10-MWT, passive ankle dorsiflexion (flexion-extension knee) and GMFT | Interactive dynamic stander using ankle  movement to play custom computer game | Session: 30 minutes, days/week: 5, weeks: 10 | Significant enhancement in GV and GMFT after training with VR.  Significant reduction in 10-MWT and mean passive ankle dorsiflexion (knee extension) after training with VR. |
| van der Krogt, Sloot, and Harlaar (2014) | Spastic and mixed | Exp: 9 participants (11.6±2.1)  Ct (healthy): 4F, 7M (10.6±2.2) | Stance time, stride length, stride time, stride width, GV, GPS (maximum ankle flexion-extension power, minimum ankle flexion-extension swing, ROM ankle flexion-extension stance/swing, knee flexion initial contact, minimum flexion-extension at knee, ROM for flexion-extension at knee, minimum flexion-extension hip, ROM flexion-extension at hip, ROM abduction-adduction at hip, ROM for pelvic tilt, ROM for pelvic obliquity), and subjective scores | Exp: Overground walking (natural, laboratory), treadmill with VR  Ct: Overground walking (natural, laboratory), treadmill with VR | - | Enhancement in GPS for Exp.  No effect on GV, stride length, cadence, stride width in Exp. |
| Burdea et al. (2013) | Spastic and mixed | 3M (10±2.6) | Dorsiflexion-plantarflexion torque, dorsiflexion initial contact, GV, Paediatric Quality of Life self-report and GMFT | Ankle training with robot-assistance and VR | Session: 40 minutes, days/week: 3, weeks: 12 | Significant enhancement in GV, Paediatric Quality of Life self-report and GMFT after training with VR. |
| Luna-Oliva et al. (2013) | Spastic and mixed | 6F, 5M (7.9±2.7) | GMFT, 10-MWT, Assessment of Motor and Process Skills, Jebsen-Taylor Test of Hand Function and Paediatric reach test | VR training with Xbox 360 Kinect | Session: 30 minutes, days/week: 2, weeks: 8 | Significant enhancement in GMFT, 10-MWT, Assessment of Motor and Process Skills and Paediatric reach test after training with VR. |
| Chen et al. (2013) | Spastic and mixed | Exp: 4F, 9M (8.7±2.1)  Ct: 5F, 9M (8.6±2.2) | GMFT, muscle strength, curl up scores, area bone mineral density and isokinetic torque | Exp: Home-based virtual cycling training  Ct: General physical exercise at home | Session: 40 minutes, days/week: 3, weeks: 12 | Significant enhancement in bone mineral density in Exp as compared to Ct. |
| Brien and Sveistrup (2011) | Spastic and mixed | 4 (16.1±4.1) | Community balance and mobility scale, GV: 6-MWT, TUG and GMFT | VR training | Session: 90 minutes, days: 5 | Significant enhancements in community balance and mobility scale, GV: 6-MWT and GMFT after virtual-reality training. |
| Exp: Experimental group, Ct: Control group, GMFT: Gross motor function test, VR: Virtual reality, 1, 2, 6, 10-MWT: 1, 2, 6, 10-metre walking test, TUG: Timed-up and go test, FRT: Functional reach test, QUEST score: Quality of Upper Extremity Skills Test, TVPS score: Test for Visual-Perceptual Skills, GV: Gait velocity, ROM: Range of motion, GPS: Gait profile score | | | | | | |

Table 4 Individual PEDro scoring for the included studies

| Studies | Eligibility criteria | Random allocation | Concealed allocation | Baseline comparability | Blind subjects | Blind therapists | Blind assessors | Adequate follow-up | Intention to treat | Between group comparison | Point estimates & variability | PEDro score |
| --- | --- | --- | --- | --- | --- | --- | --- | --- | --- | --- | --- | --- |
| Levac et al. (2018) | Y | 1 | 0 | 1 | 0 | 0 | 0 | 1 | 0 | 1 | 1 | 5 |
| Gagliardi et al. (2018) | Y | 1 | 0 | 1 | 0 | 1 | 0 | 1 | 0 | 1 | 1 | 6 |
| Chiu et al. (2018) | Y | 1 | 0 | 1 | 0 | 0 | 0 | 1 | 0 | 1 | 1 | 5 |
| Jung et al. (2018) | Y | 1 | 0 | 1 | 0 | 0 | 0 | 0 | 0 | 1 | 1 | 4 |
| Sajan et al. (2017) | Y | 1 | 0 | 1 | 1 | 1 | 1 | 1 | 0 | 1 | 1 | 8 |
| Tarakci et al. (2016) | Y | 1 | 1 | 1 | 1 | 0 | 1 | 0 | 0 | 1 | 1 | 7 |
| Mitchell et al. (2016) | Y | 1 | 1 | 1 | 1 | 0 | 1 | 0 | 0 | 1 | 1 | 7 |
| Cho et al. (2016) | Y | 1 | 1 | 1 | 1 | 0 | 1 | 0 | 0 | 1 | 1 | 7 |
| AlSaif and Alsenany (2015) | Y | 1 | 0 | 1 | 0 | 0 | 0 | 1 | 0 | 1 | 1 | 5 |
| Collange Grecco et al. (2015) | Y | 1 | 1 | 1 | 1 | 1 | 1 | 1 | 0 | 1 | 1 | 9 |
| Curtis et al. (2014) | Y | 1 | 0 | 1 | 0 | 1 | 0 | 1 | 0 | 1 | 1 | 6 |
| van der Krogt et al. (2014) | Y | 1 | 0 | 1 | 0 | 0 | 0 | 1 | 0 | 1 | 1 | 5 |
| Burdea et al. (2013) | Y | 1 | 0 | 1 | 0 | 0 | 0 | 0 | 0 | 1 | 1 | 4 |
| Luna-Oliva et al. (2013) | Y | 1 | 0 | 1 | 0 | 0 | 0 | 1 | 0 | 1 | 1 | 5 |
| Chen et al. (2013) | Y | 1 | 0 | 1 | 0 | 0 | 0 | 1 | 0 | 1 | 1 | 5 |
| Brien and Sveistrup (2011) | Y | 1 | 0 | 1 | 0 | 0 | 0 | 0 | 0 | 1 | 1 | 4 |

**Gait velocity**


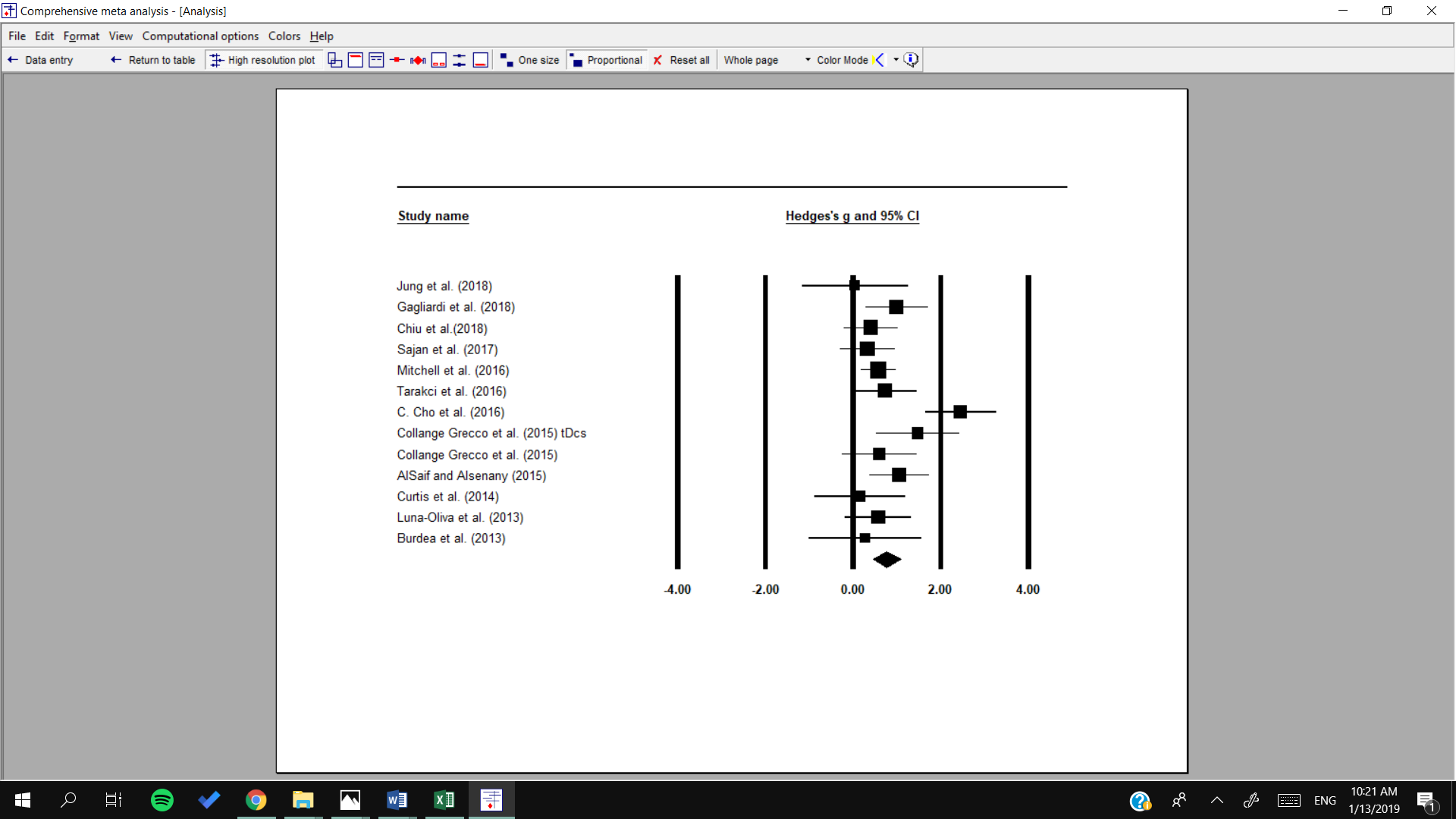


Figure 1 Forest plot illustrating individual studies evaluating the effects Virtual reality training on gait velocity amongst children with cerebral palsy (only training studies). Weighted effect sizes; Hedge’s g (boxes) and 95% C.I (whiskers) are presented, demonstrating repositioning errors for individual studies. The (Diamond) represents pooled effect sizes and 95% CI. A negative effect size indicated reduction in gait velocity; a positive effect size indicated enhancement in gait velocity. (tDcs: transcranial direct current)


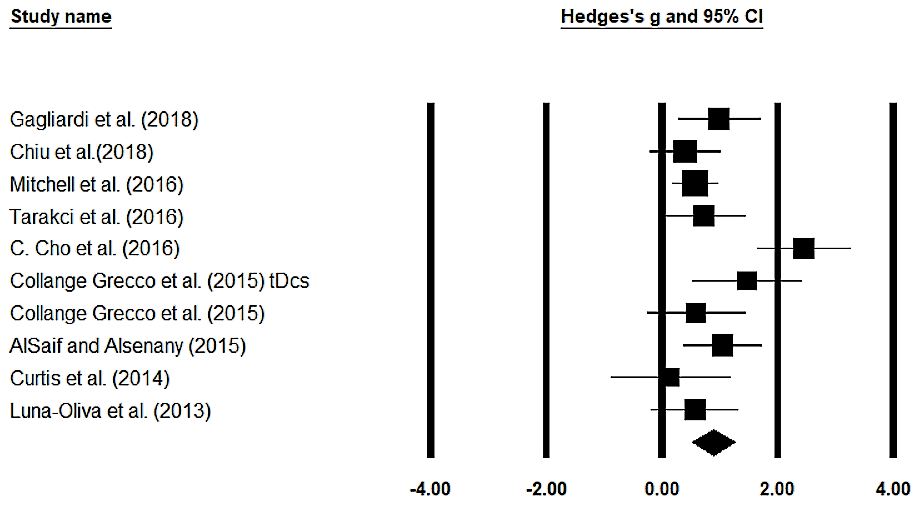


Figure 2 Forest plot illustrating individual studies evaluating the effects Virtual reality training on gait velocity amongst children with cerebral palsy (20-30 minutes training).


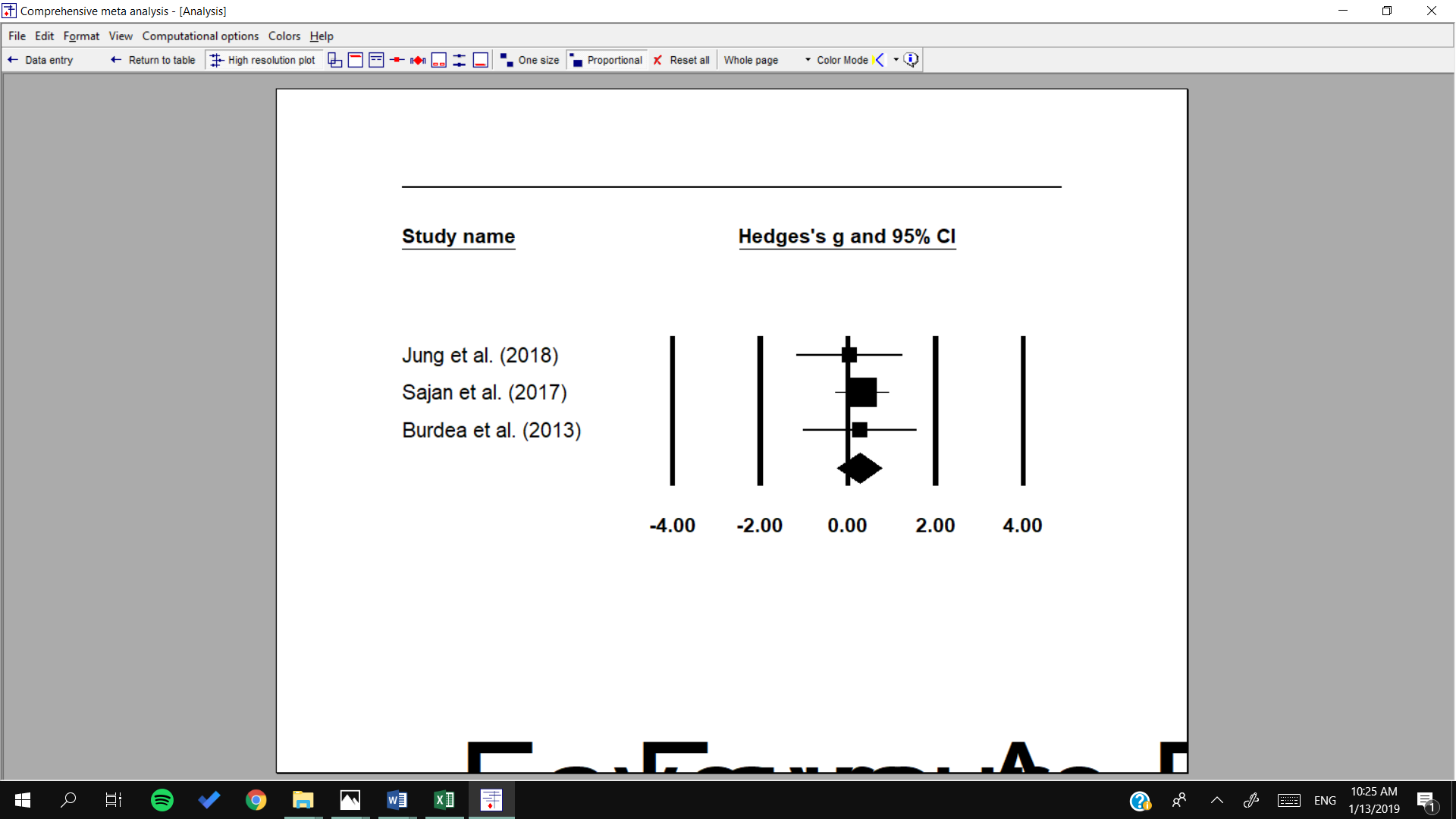


Figure 3 Forest plot illustrating individual studies evaluating the effects Virtual reality training on gait velocity amongst children with cerebral palsy (40-45 minutes training).


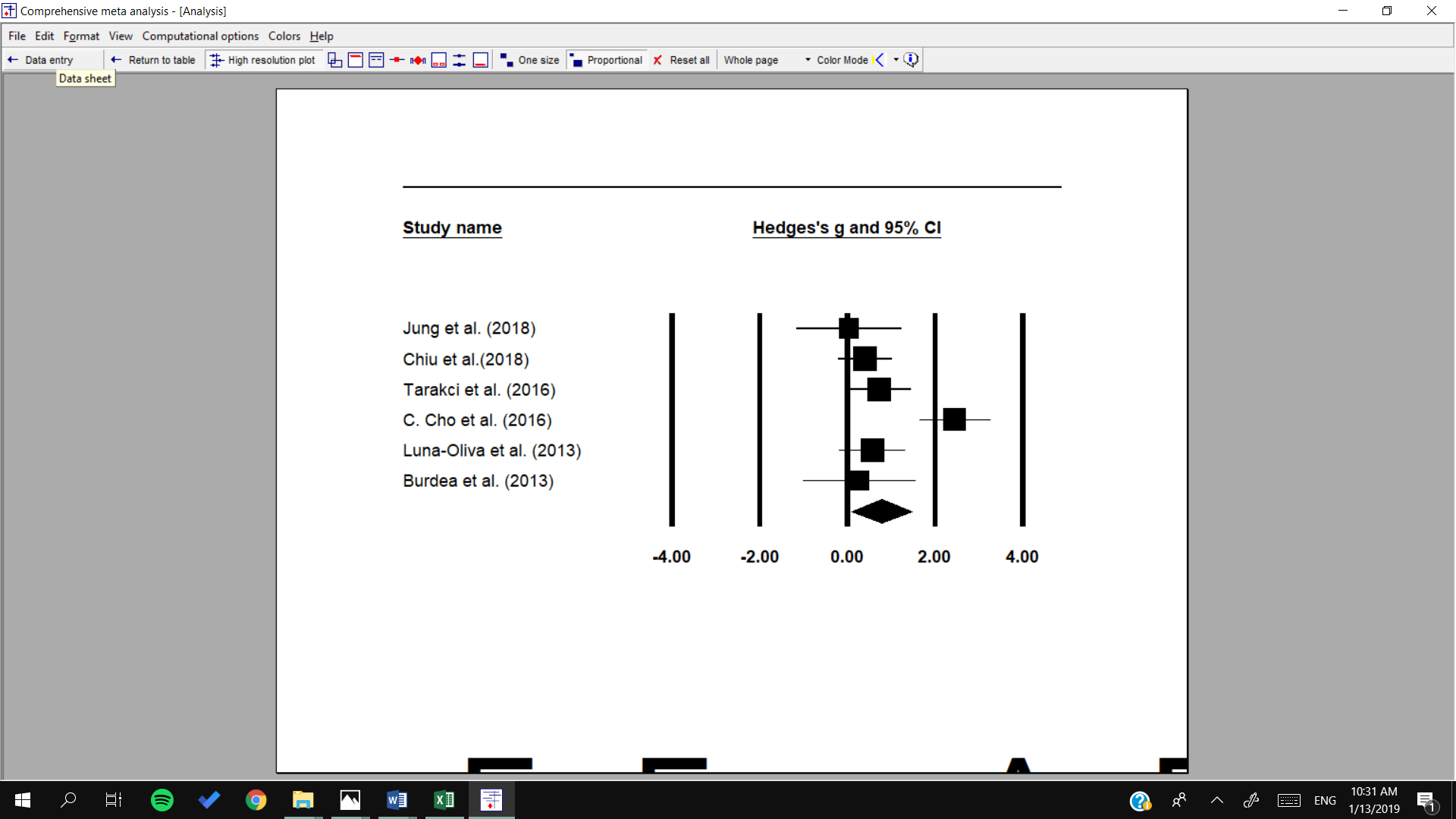


Figure 4 Forest plot illustrating individual studies evaluating the effects Virtual reality training on gait velocity amongst children with cerebral palsy (≤4 sessions per week).


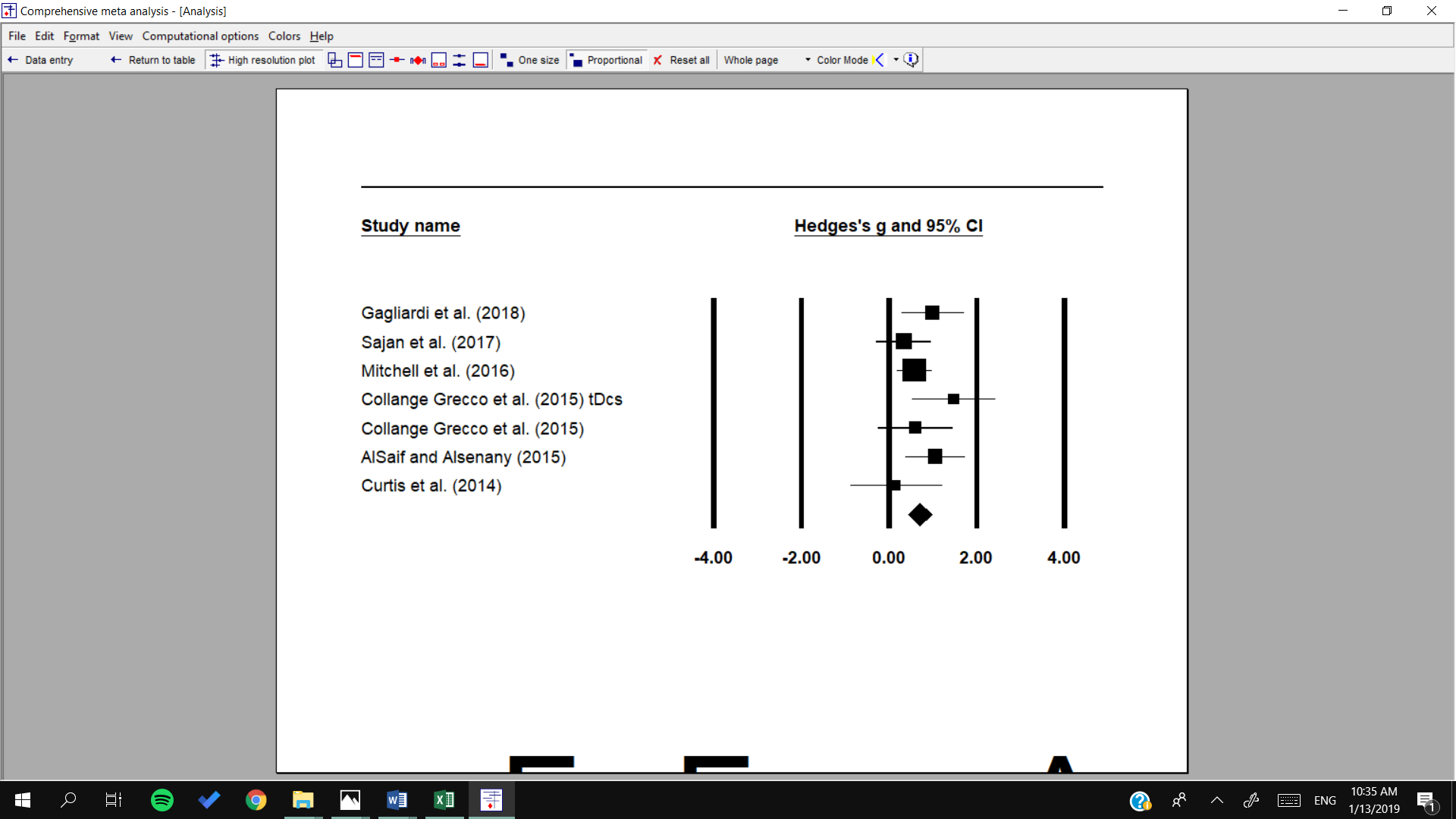


Figure 5 Forest plot illustrating individual studies evaluating the effects Virtual reality training on gait velocity amongst children with cerebral palsy (≥5 sessions per week).


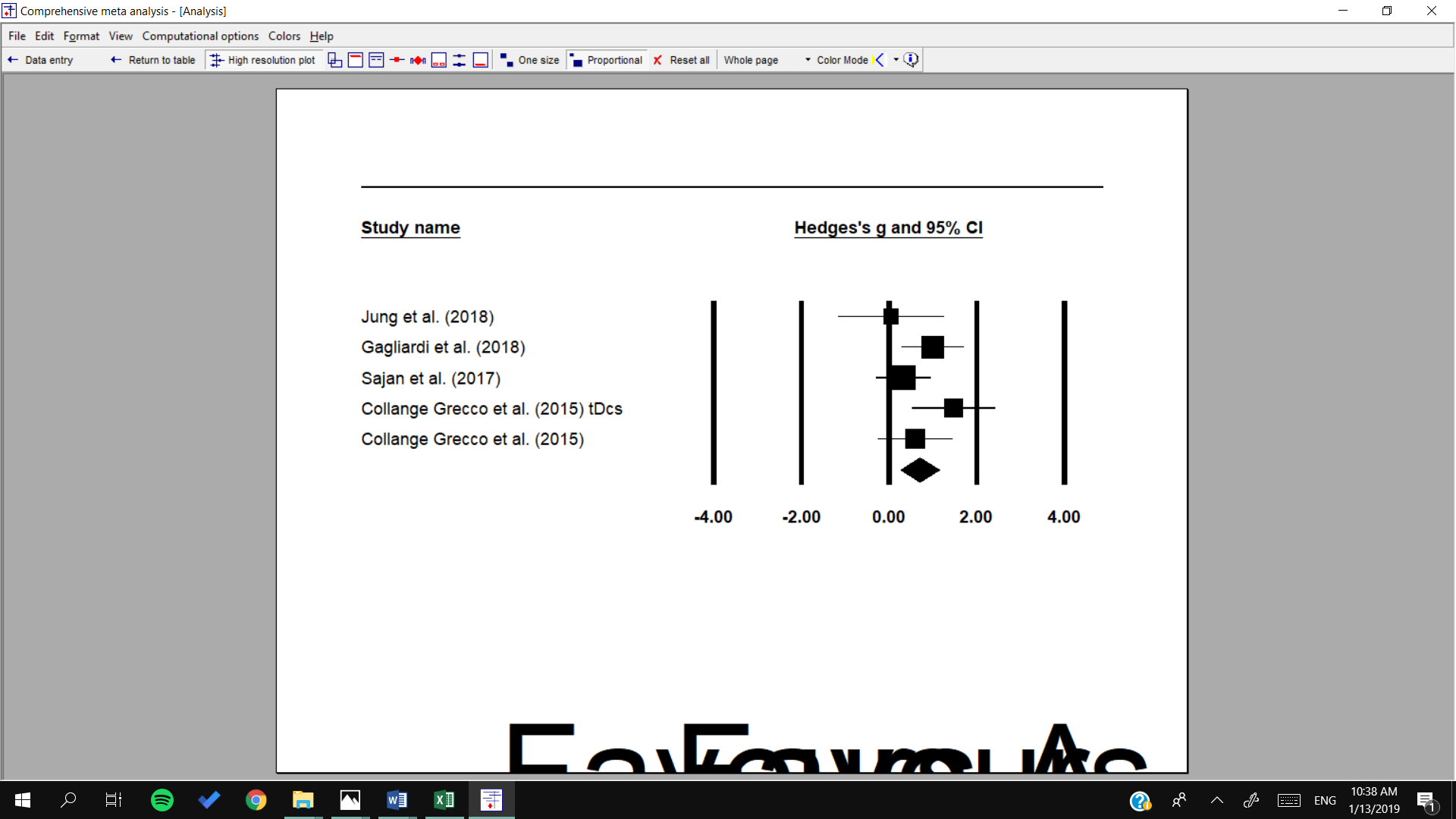


Figure 6 Forest plot illustrating individual studies evaluating the effects Virtual reality training on gait velocity amongst children with cerebral palsy (≥8 weeks).

Figure 7 Forest plot illustrating individual studies evaluating the effects Virtual reality training on gait velocity amongst children with cerebral palsy (≤7 weeks).

**Stride length**


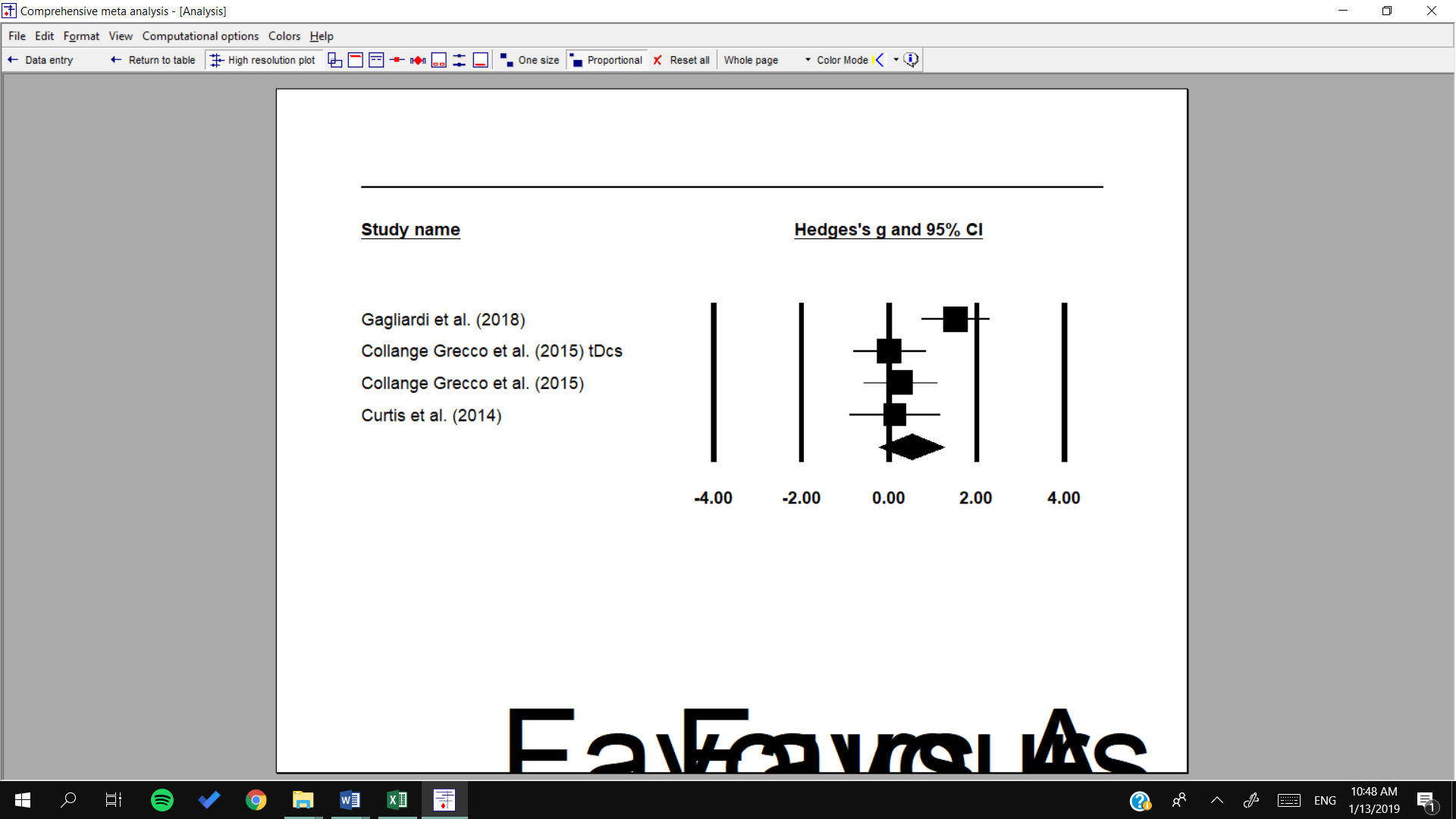


Figure 8 Forest plot illustrating individual studies evaluating the effects of virtual reality training on stride length amongst children with cerebral palsy (only training).


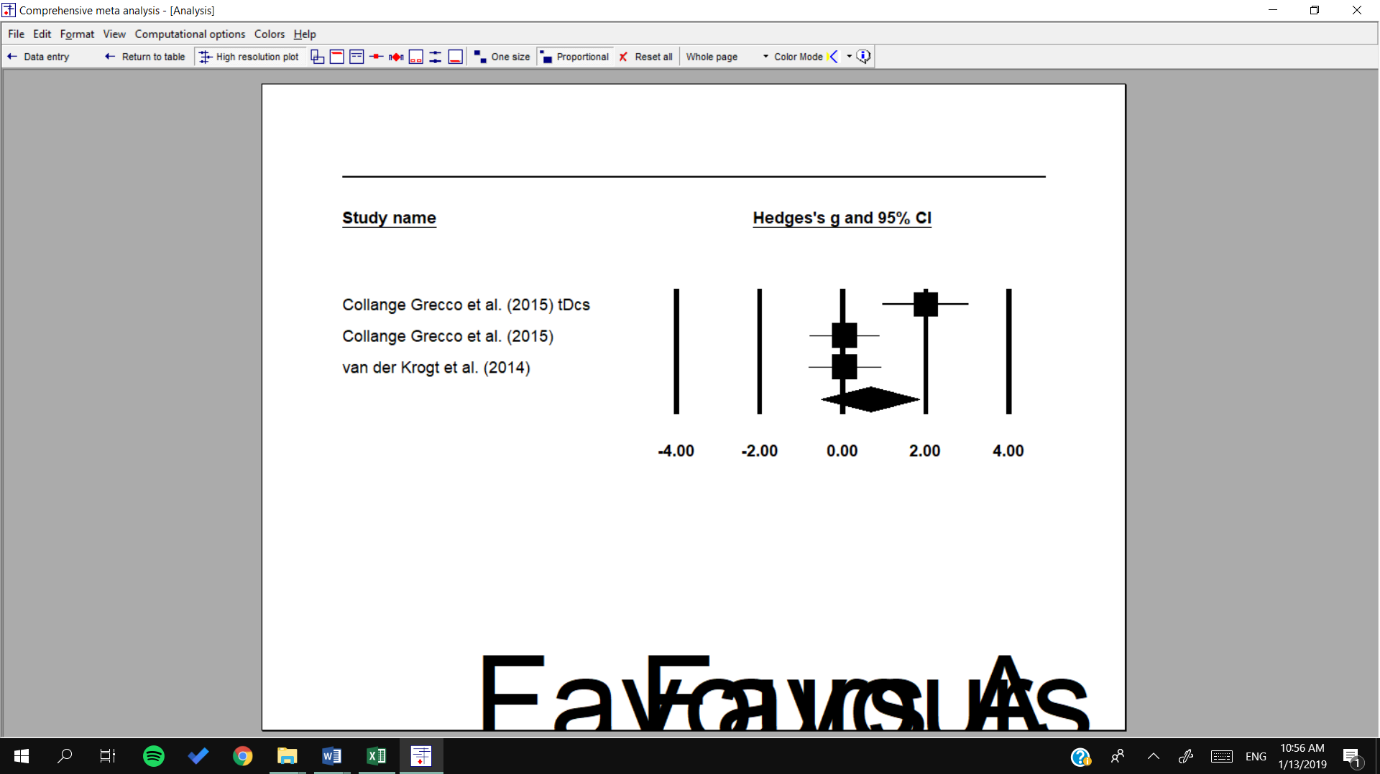


Figure 9 Forest plot illustrating individual studies evaluating the effects of virtual reality training on cadence amongst children with cerebral palsy.

**Stride width**


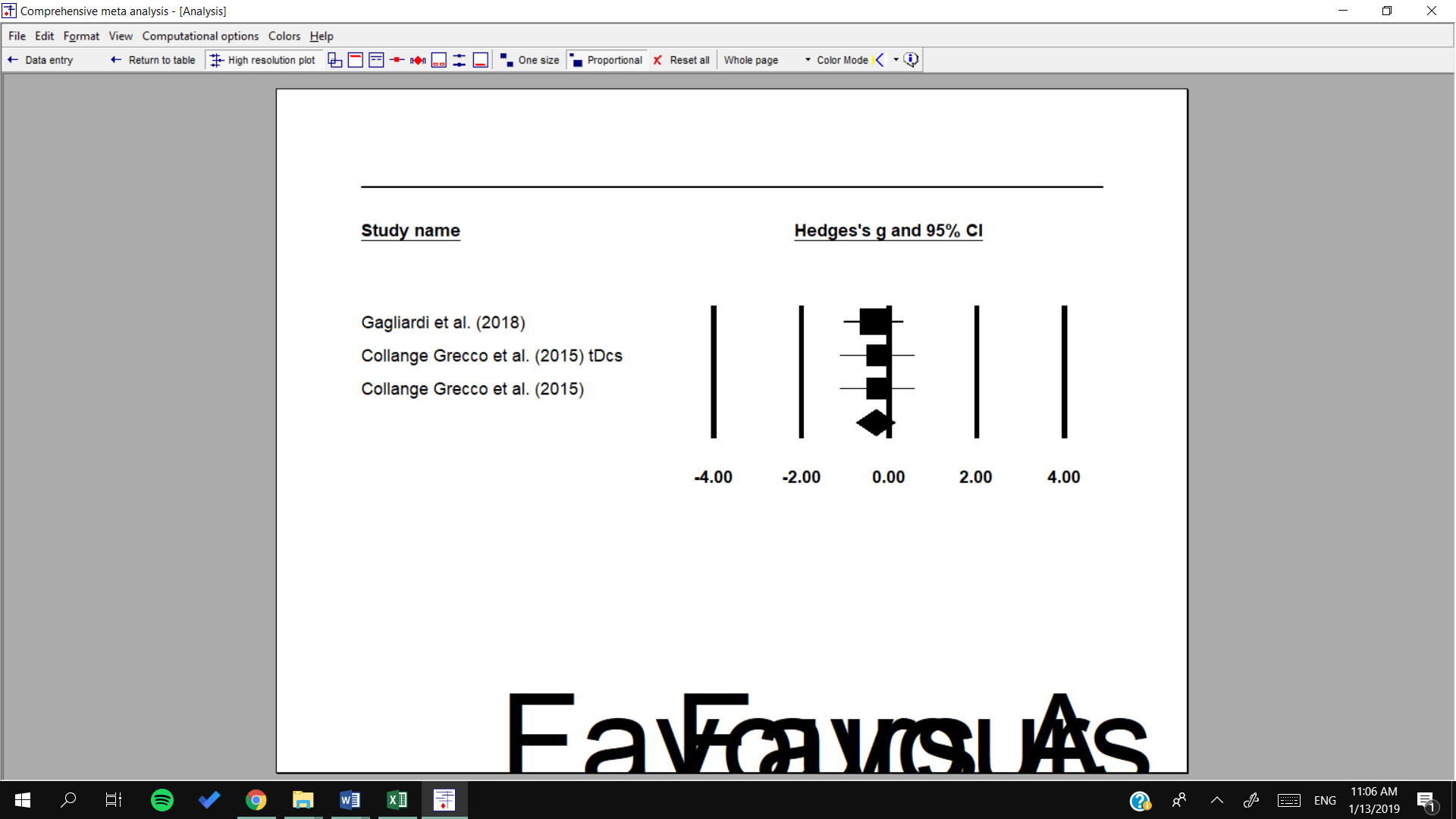


Figure 10 Forest plot illustrating individual studies evaluating the effects of virtual reality training on stride width amongst children with cerebral palsy (only training).

**Gross motor function test**


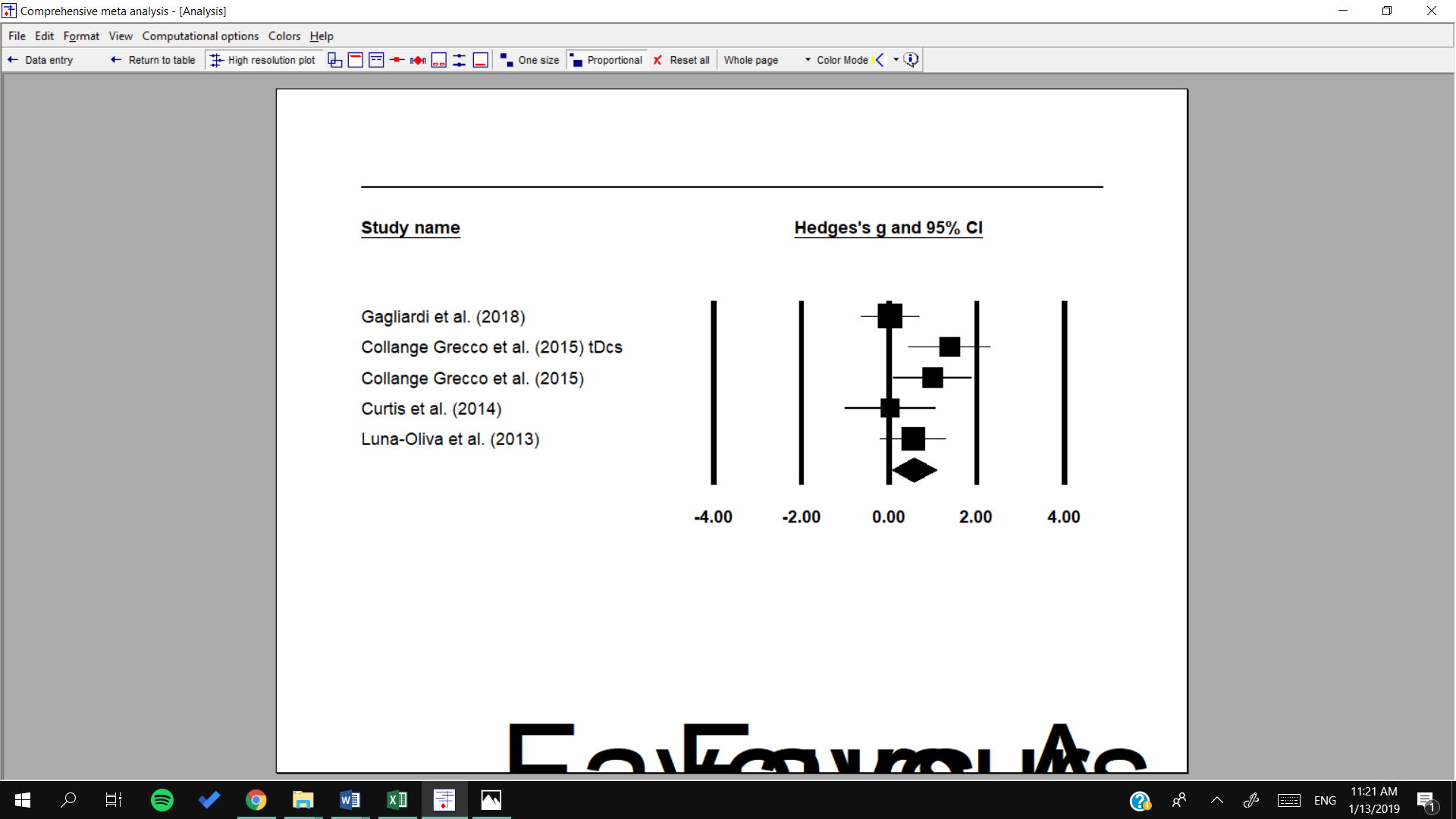


Figure 11 Forest plot illustrating individual studies evaluating the effects Virtual reality training on Gross motor function test amongst children with cerebral palsy (20-30 minutes training).


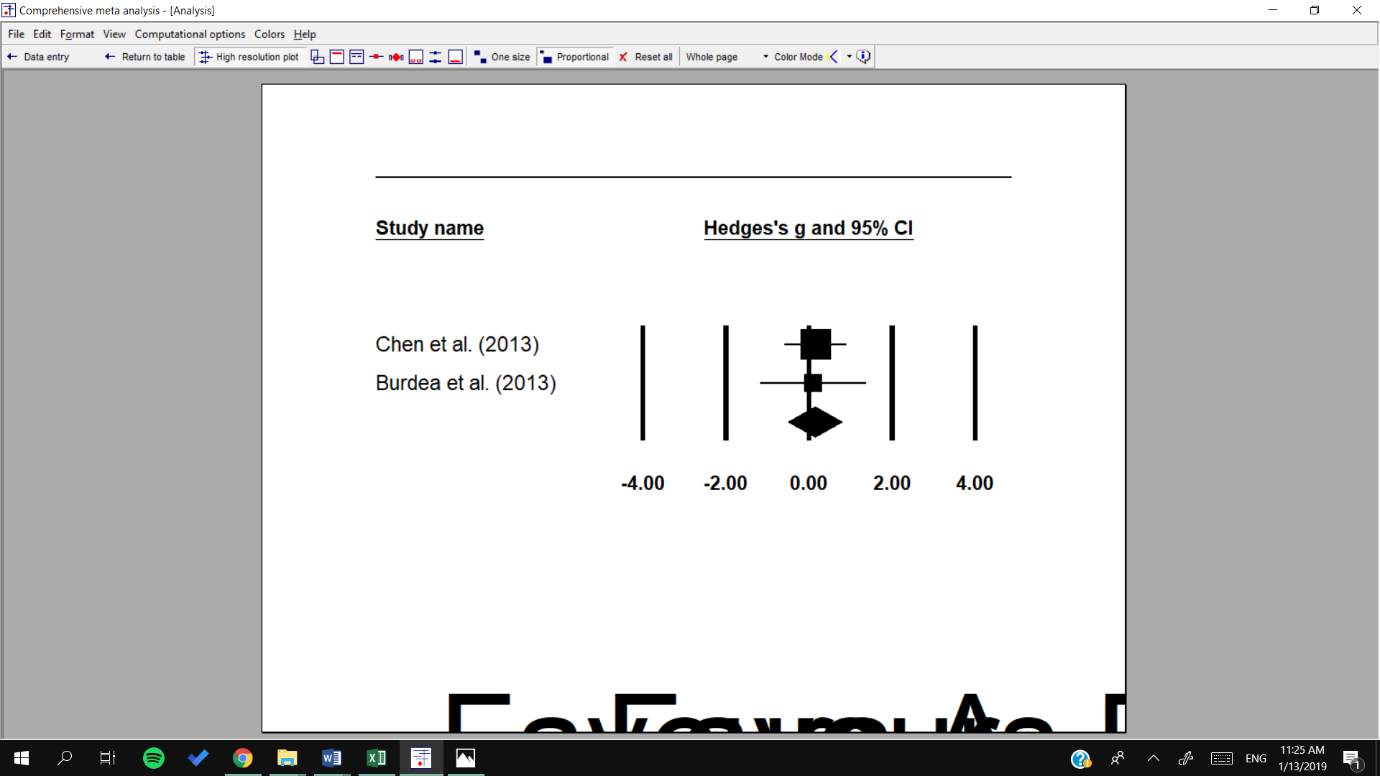


Figure 12 Forest plot illustrating individual studies evaluating the effects Virtual reality training on Gross motor function test amongst children with cerebral palsy (40-45 minutes training).

**References**

AlSaif, A. A., & Alsenany, S. (2015). Effects of interactive games on motor performance in children with spastic cerebral palsy. *Journal of physical therapy science, 27*(6), 2001-2003.

Borenstein, M. (2005). Software for publication bias. *Publication bias in meta-analysis: Prevention, assessment and adjustments*, 193-220.

Borenstein, M., Hedges, L. V., Higgins, J., & Rothstein, H. R. (2010). A basic introduction to fixed‐effect and random‐effects models for meta‐analysis. *Research synthesis methods, 1*(2), 97-111.

Brien, M., & Sveistrup, H. (2011). An intensive virtual reality program improves functional balance and mobility of adolescents with cerebral palsy. *Pediatric Physical Therapy, 23*(3), 258-266.

Burdea, G. C., Cioi, D., Kale, A., Janes, W. E., Ross, S. A., & Engsberg, J. R. (2013). Robotics and gaming to improve ankle strength, motor control, and function in children with cerebral palsy-a case study series. *IEEE Transactions on Neural Systems and Rehabilitation Engineering, 21*(2), 165-173.

Chen, C.-L., Chen, C.-Y., Liaw, M.-Y., Chung, C.-Y., Wang, C.-J., & Hong, W.-H. (2013). Efficacy of home-based virtual cycling training on bone mineral density in ambulatory children with cerebral palsy. *Osteoporosis international, 24*(4), 1399-1406.

Chiu, H.-C., Ada, L., & Lee, S.-D. (2018). Balance and mobility training at home using Wii Fit in children with cerebral palsy: a feasibility study. *BMJ open, 8*(5), e019624.

Cho, C., Hwang, W., Hwang, S., & Chung, Y. (2016). Treadmill training with virtual reality improves gait, balance, and muscle strength in children with cerebral palsy. *The Tohoku journal of experimental medicine, 238*(3), 213-218.

Cohen, J. (1988). *Statistical power analysis for the behavioral sciences* (2nd ed.). Hillsdale, NJ: L, Erlbaum Associates.

Collange Grecco, L. A., de Almeida Carvalho Duarte, N., Mendonça, M. E., Galli, M., Fregni, F., & Oliveira, C. S. (2015). Effects of anodal transcranial direct current stimulation combined with virtual reality for improving gait in children with spastic diparetic cerebral palsy: a pilot, randomized, controlled, double-blind, clinical trial. *Clinical rehabilitation, 29*(12), 1212-1223.

Cooper, H., Hedges, L. V., & Valentine, J. C. (2009). *The handbook of research synthesis and meta-analysis*: Russell Sage Foundation.

Cumming, G. (2013). *Understanding the new statistics: Effect sizes, confidence intervals, and meta-analysis*: Routledge.

Curtis, D. J., Bencke, J., & Mygind, B. (2014). The effect of training in an interactive dynamic stander on ankle dorsiflexion and gross motor function in children with cerebral palsy. *Developmental neurorehabilitation, 17*(6), 393-397.

de Morton, N. A. (2009). The PEDro scale is a valid measure of the methodological quality of clinical trials: a demographic study. *Australian Journal of Physiotherapy, 55*(2), 129-133.

Elkins, M. R., Herbert, R. D., Moseley, A. M., Sherrington, C., & Maher, C. (2010). Rating the Quality of Trials in Systematic Reviews of Physical Therapy Interventions. *Cardiopulmonary Physical Therapy Journal, 21*(3), 20-26.

Gagliardi, C., Turconi, A. C., Biffi, E., Maghini, C., Marelli, A., Cesareo, A., . . . Panzeri, D. (2018). Immersive virtual reality to improve walking abilities in cerebral palsy: a pilot study. *Annals of biomedical engineering*, 1-9.

Gasq, D., Le Dean, Y., Labrunee, M., Castel-Lacanal, E., Terracol, C., de Boissezon, X., & Marque, P. (2017). Between and within-day reliability of spatiotemporal gait parameters following stroke: Why measurement at maximal gait speed is required? *Annals of Physical and Rehabilitation Medicine, 60*, e4. doi:<https://doi.org/10.1016/j.rehab.2017.07.023>

Hanna, S. E., Bartlett, D. J., Rivard, L. M., & Russell, D. J. (2008). Reference curves for the Gross Motor Function Measure: percentiles for clinical description and tracking over time among children with cerebral palsy. *Physical therapy, 88*(5), 596-607.

Higgins, J. P., & Green, S. (2011). *Cochrane handbook for systematic reviews of interventions* (Vol. 4): John Wiley & Sons.

Higgins, J. P. T., Thompson, S. G., Deeks, J. J., & Altman, D. G. (2003). Measuring inconsistency in meta-analyses. *BMJ : British Medical Journal, 327*(7414), 557-560.

Jung, S.-H., Song, S.-H., Kim, S.-D., Lee, K., & Lee, G.-C. (2018). Does virtual reality training using the Xbox Kinect have a positive effect on physical functioning in children with spastic cerebral palsy? A case series. *Journal of pediatric rehabilitation medicine, 11*(2), 95-101.

Levac, D., McCormick, A., Levin, M. F., Brien, M., Mills, R., Miller, E., & Sveistrup, H. (2018). Active video gaming for children with cerebral palsy: Does a clinic-based virtual reality component offer an additive benefit? A pilot study. *Physical & occupational therapy in pediatrics, 38*(1), 74-87.

Luna-Oliva, L., Ortiz-Gutiérrez, R. M., Cano-de la Cuerda, R., Piédrola, R. M., Alguacil-Diego, I. M., Sánchez-Camarero, C., & Martínez Culebras, M. d. C. (2013). Kinect Xbox 360 as a therapeutic modality for children with cerebral palsy in a school environment: a preliminary study. *NeuroRehabilitation, 33*(4), 513-521.

Mitchell, L. E., Ziviani, J., & Boyd, R. N. (2016). A randomized controlled trial of web‐based training to increase activity in children with cerebral palsy. *Developmental Medicine & Child Neurology, 58*(7), 767-773.

Moher, D., Liberati, A., Tetzlaff, J., Altman, D. G., & Group, P. (2009). Preferred reporting items for systematic reviews and meta-analyses: the PRISMA statement. *PLoS medicine, 6*(7), e1000097.

Sajan, J. E., John, J. A., Grace, P., Sabu, S. S., & Tharion, G. (2017). Wii-based interactive video games as a supplement to conventional therapy for rehabilitation of children with cerebral palsy: a pilot, randomized controlled trial. *Developmental neurorehabilitation, 20*(6), 361-367.

Sue, D., & Richard, T. (2000). Trim and Fill: A Simple Funnel-Plot–Based Method of Testing and Adjusting for Publication Bias in Meta-Analysis. *Biometrics, 56*(2), 455-463. doi:doi:10.1111/j.0006-341X.2000.00455.x

Tarakci, D., Ersoz Huseyinsinoglu, B., Tarakci, E., & Razak Ozdincler, A. (2016). Effects of Nintendo Wii‐Fit® video games on balance in children with mild cerebral palsy. *Pediatrics international, 58*(10), 1042-1050.

Teasell, R. (2008). Evidence-based review of stroke rehabilitation (EBRSR).

van der Krogt, M. M., Sloot, L. H., & Harlaar, J. (2014). Overground versus self-paced treadmill walking in a virtual environment in children with cerebral palsy. *Gait & posture, 40*(4), 587-593.
